# Data-driven flood susceptibility assessment using hybrid machine learning and optimization techniques: case of the Sedrata Watershed, NE Algeria

**DOI:** 10.1038/s41598-026-43262-9

**Published:** 2026-04-04

**Authors:** Elhadi Mechentel, Sabri Dairi, Abdelouahab Lefkir, Saeid Eslamian, Habib Abida, Yassine Djebbar

**Affiliations:** 1 Department of Hydraulics, Badji Mokhtar University, Annaba, Algeria; 2 Laboratory of Research Infra-Res, Mohamed Cherif Messaadia, Souk Ahras, Algeria; 3 École Nationale Supérieure des Travaux Publics (ENSTP), Kouba, Algiers Algeria 32,; 4 Department of Water Science and Engineering, College of Agriculture, Isfahan University of Technology, Isfahan, Iran; 5 GEOMODELE Laboratory, Faculty of Sciences, University of Sfax, Sfax, Tunisia

**Keywords:** Flood susceptibility mapping, Machine learning, Metaheuristic optimization, Satellite imagery, Sedrata Watershed, Environmental sciences, Hydrology, Natural hazards

## Abstract

Flooding is one of the most disastrous natural hazards around the globe, causing enormous ecological and socio-economic losses; therefore, reliable assessment tools are required for informed risk management. This research proposes a hybrid flood susceptibility modeling framework that incorporates the Random Forest Regressor (RFR) model coupled with three metaheuristic optimization algorithms, namely Grasshopper Optimization Algorithm (GOA), Salp Swarm Algorithm (SSA), and Ant Colony Optimization (ACO). The proposed approach is applied to the Sedrata Watershed, Northeastern Algeria, using satellite data along with twelve physiographic and environmental conditioning factors, including slope, rainfall, land use, drainage density, curvature, convexity, and aspect. A total of 317 flood locations detected from Sentinel-1 imagery were used for training and validation of the hybrid models. In this respect, the Weight of Evidence and Geographically Weighted Regression methods have been applied to investigate the influence of each conditioning factor. These hybrid models significantly enhanced predictive performance when compared to the standalone RFR model, showing Area Under Curve (AUC) values of 0.928, 0.925, and 0.920 for RFR-ACO, RFR-SSA, and RFR-GOA models respectively, against 0.904 obtained by the baseline model. The spatial mapping showed that about 26% of the study area has high to very high flood susceptibility. These findings confirm the strong potential of combining Machine Learning and metaheuristic optimization in view of enhancing the predictive performance offlood susceptibility maps. The proposed framework offers an interpretable decision-support tool for sustainable flood risk management and land use planning in the Sedrata region.

## Introduction

Floods are among the most frequent and devastating natural hazards, exerting major impacts on human life, economies, and ecosystems^1,2^. In the period between 1975 and 2022, more than 5,500 flood events were recorded worldwide, with approximately 70% of them originating from rivers,these have thus far affected over 2.5 billion people and caused more than 224,000 fatalities^3–5^. During the last five decades alone, flooding has accounted for more than half of the overall damage produced by natural disasters^6^. Very recently, tragic events in Germany in 2021, in Pakistan and Iran in 2022, and in Brazil and France in 2024 brought into light a scenario of an increasing intensity and recurrence under a changing climate^7,8^. There is further enhancement of vulnerability due to rapid urbanization, population growth, land-use changes, and often inappropriate planning, particularly in the areas of rapid development^9,10^.

In this respect, flood susceptibility mapping (FSM) has become an essential tool to identify areas prone to risk and serve land-use planning, optimize preparedness, and support early warning systems^11–13^. Early approaches to FSM have been essentially statistical,for instance, Frequency Ratio (FR), Weight of Evidence (WoE), and Conditional Function (CF)methods have gained widespread application due to their interpretability^14–17^. However, these approaches cannot capture nonlinear relationships and complex interactions among conditioning factors^18,19^. On the other hand, methods such as Analtical Hierarchy Process (AHP), Technique for Order Preference by Similarity to Ideal Solution(TOPSIS), and Best–Worst are sensitive to subjective weighting^20–22^.

Recent advances in Machine Learning (ML) and Deep Learning (DL) have significantly enhanced FSM by exploiting large and heterogeneous datasets to model complex spatial and nonlinear dependencies. ML Algorithms such as Random Forest (RF), Support Vector Machine (SVM), Decision Tree (DT), K-Nearest Neighbors (KNN), Naive Bayes, Extreme Gradient Boosting (XGBoost), CatBoost, and, on the Deep Learning side, Artificial Neural Networks (ANN), Convolutional Neural Networks (CNN), Recurrent Neural Networks (RNN), and Long short-Term Memory (LSTM), reached high performance in various hydrological contexts^23,24,24–33^.

In parallel, remote sensing advances have transformed flood detection and FSM modeling. Synthetic Aperture Radar (SAR) sensors, especially Sentinel-1, offer all-weather, day-and-night observations that become important during cloudy and rainy conditions, thus enabling the robust detection of inundated areas by comparing pre- and post-event images^34–37^. The integration of Sentinel-1 data with other satellite sources, such as Landsat-8, further enhances spatiotemporal coverage for flood modeling^38,39^.

Despite these developments, a number of challenges still remain for the development of truly operational models, including the choice of appropriate algorithms and their settings, quality and feature engineering of data, interpretability of models, and particularly fine-tuning of hyperparameters, often computationally expensive and time-consuming when done manually^40–42^. As a response, hybrid methods coupling ML/DL with metaheuristic optimization have recently come to prominence because they effectively balance the exploration–exploitation trade-off of the solution space, avoid the entrapment in local optima, and include intrinsic parallelization capabilities^43–47^.

A large number of studies have successfully coupled metaheuristic algorithms, such as Genetic Alghorithms (GA), Particle Swarm Optimization (PSO), Ant Colony Optimization (ACO), Grey Wolf Optimizer (GWO), and Harmony Search (HS), with ML models including ANN, SVR, ANFIS(Adaptive Neuro-Fuzzy Inference System), DNN, and XGBoost, reporting substantial performance improvements compared to the nonoptimized models^48–50^. However, most of the literature so far appears to be focused on specific combinations of algorithms and mostly lacks full integration with multi-source geospatial data, advanced optimization strategies, and SARbased flood detection, which limits the spatial generalizability and robustness of susceptibility maps^51,52^.

The present study was conducted within the Sedrata Watershed, situated in the Souk-Ahras Province of Northeastern Algeria, in proximity to the Tunisian border. This area lies within the transition zone between a semi-arid and a sub-humid climate in the Mediterranean region. Because of the irregular distribution of rainfall, with short but intense precipitation events, hydrological risks become extremely high for the occurrence of flash flooding. This watershed was chosen based on a high frequency of recurrent flood events that have historically caused significant environmental degradation, infrastructure damage, and socio-economic disruption.

To deal with these challenges, the current study proposes a methodological framework that seeks to enhance the accuracy and reliability of flood susceptibility mapping. The developed approach is characterized by the combined integration of various state-of-the-art tools and techniques within a single workflow: the exploitation of multisource geospatial data, Sentinel-1 SAR imagery for detecting floods, and the hybrid Machine Learning approach. In this respect, the predictive model relies on Random Forest Regression (RFR) as the base algorithm whose performance is improved through the use of three state-of-the-art population-based metaheuristic algorithms, namely Grasshopper Optimization Algorithm (GOA), Salp Swarm Algorithm (SSA), and Ant Colony Optimization (ACO), capable of fine-tuning model parameters and, thus, substantially improving the predictive accuracy. Also, Weight of Evidence (WoE), analysis of multicollinearity, and Geographically Weighted Regression (GWR) were performed as some analytical tools to investigate the spatial variability and statistical relevance of the conditioning factors.

Previous studies dealing with flood susceptibility modeling consistently identify ensemble tree-based methods, particularly Random Forest (RF), as among the most robust and reliable machine learning approaches. Since its introduction by Breiman^53^, RF has been widely applied in hydrology because of its ability to capture nonlinear relationships, handle multicollinearity, and maintain strong generalization under noisy or limited data conditions^32,33,54,55^. Comparative studies conducted in different climatic settings, including Europe, Asia, and North Africa, report that RF-based models often outperform or perform on par with SVM, ANN, and logistic regression in flood susceptibility mapping^24,27,56^. Furthermore, both early and recent hydrological studies demonstrate that coupling machine learning models with population-based metaheuristic optimization algorithms significantly enhances predictive accuracy and model stability compared to non-optimized configurations^57–59^. Recent works have specifically reported clear performance gains when Random Forest, ANN and SVR models are tuned via metaheuristics such as GA, PSO or GWO, rather than relying on default or manually selected hyperparameters^60–64^.

Complementing these case studies, recent review articles, including the systematic review of Pourzangbar et al^13^., underline the central role of rigorous model selection, systematic hyperparameter optimization and integration of multi‑source geospatial data in state‑of‑the‑art hydrological ML applications, thereby providing a strong conceptual basis for metaheuristic‑optimized RF‑type models in susceptibility analyses^65–71^. Guided by this recent literature, the present study combines RFR with three well‑established population‑based metaheuristic algorithms, GOA^72^, SSA^73^ and ACO^74^, to perform systematic hyperparameter optimization instead of ad‑hoc calibration, with the explicit objective of obtaining a more accurate and generalizable flood susceptibility model for the SedrataWatershed.

Although the combined use of satellite data and machine learning is now well established in flood susceptibility mapping,comparative optimization strategies remain limited in semi-arid Mediterranean regions and North Africa. Accordingly, this study is mainly interested in establishing a comprehensive and operational framework tailored to the Sedrata Watershed and similar semi-arid watersheds in North Africa, where flood susceptibility remains insufficiently documented despite recurrent damaging events and pronounced socio-economic vulnerability. Applied to a semi-arid to sub-humid North African watershed, this integrated framework constitutes one of the first SAR-driven, metaheuristic-optimized RFR implementations for flood susceptibility mapping in Algeria and yields a transferable, statistically grounded decision-support tool for land-use planning, risk management, and climate change adaptation in similarly exposed Mediterranean environments.

## Material and methods

### Study area

**Fig. 1:**
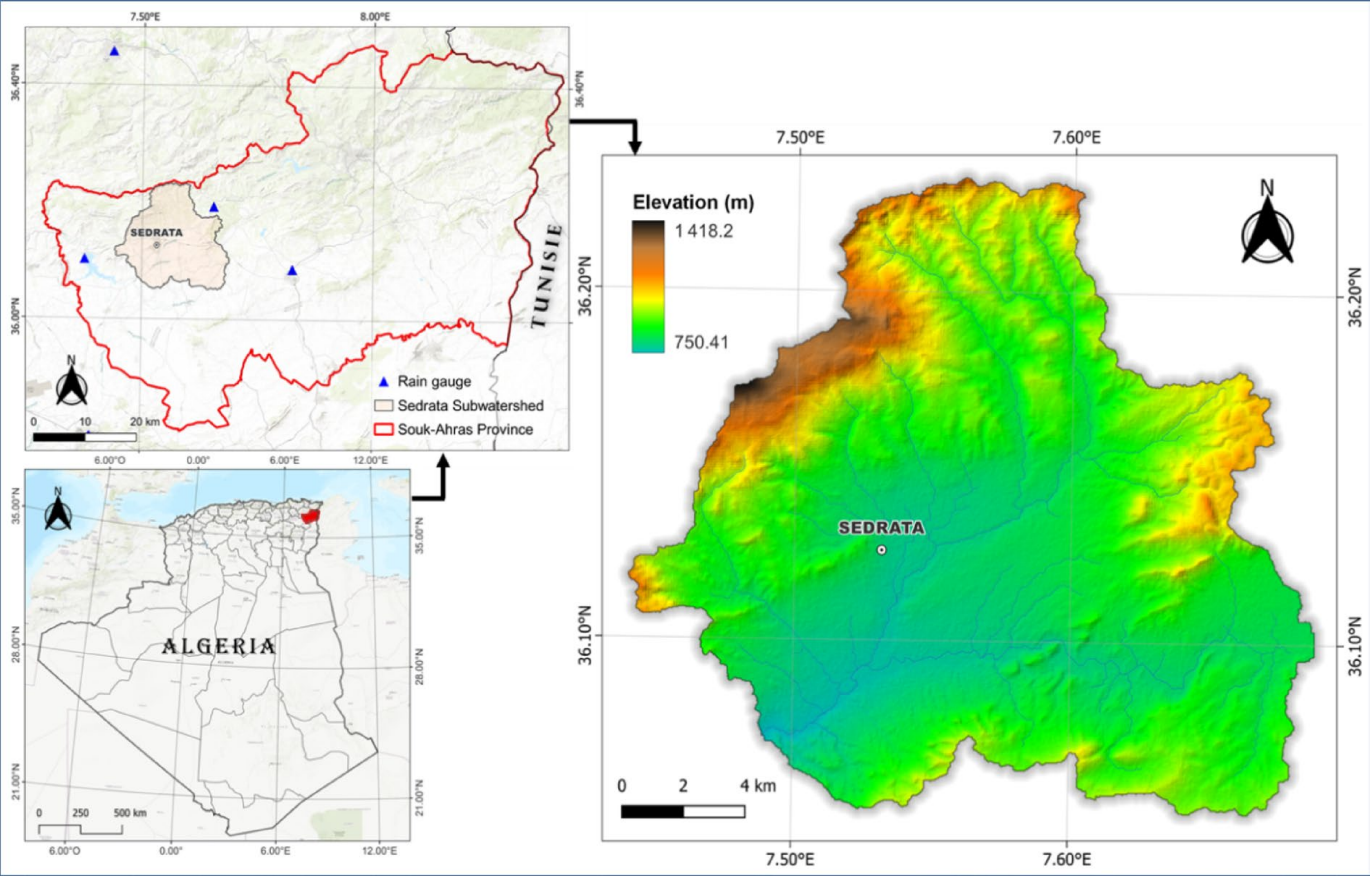
Geographical setting of the study area.

SedrataWatershed is located in the Souk-Ahras Province, Northeastern Algeria, near the Tunisian border (Fig. 1). Delineated using ArcGIS, it extends approximately between latitudes 36°12′ N and 36°03′ N and longitudes 7°26′ E and 7°40′ E. The elevation ranges from 750 m to over 1,418 m above sea level, covering a total area of about 285 km^2^. The region’s climate lies within the semi-arid to sub-humid bioclimatic zones and is characterized by short but intense rainfall events. Land use is predominantly agricultural, with significant urban settlements. This watershed was selected for study because of the recurrent floods that frequently hit the region, resulting in considerable environmental and socio-economic impacts.

## Methodology

**Fig. 2:**
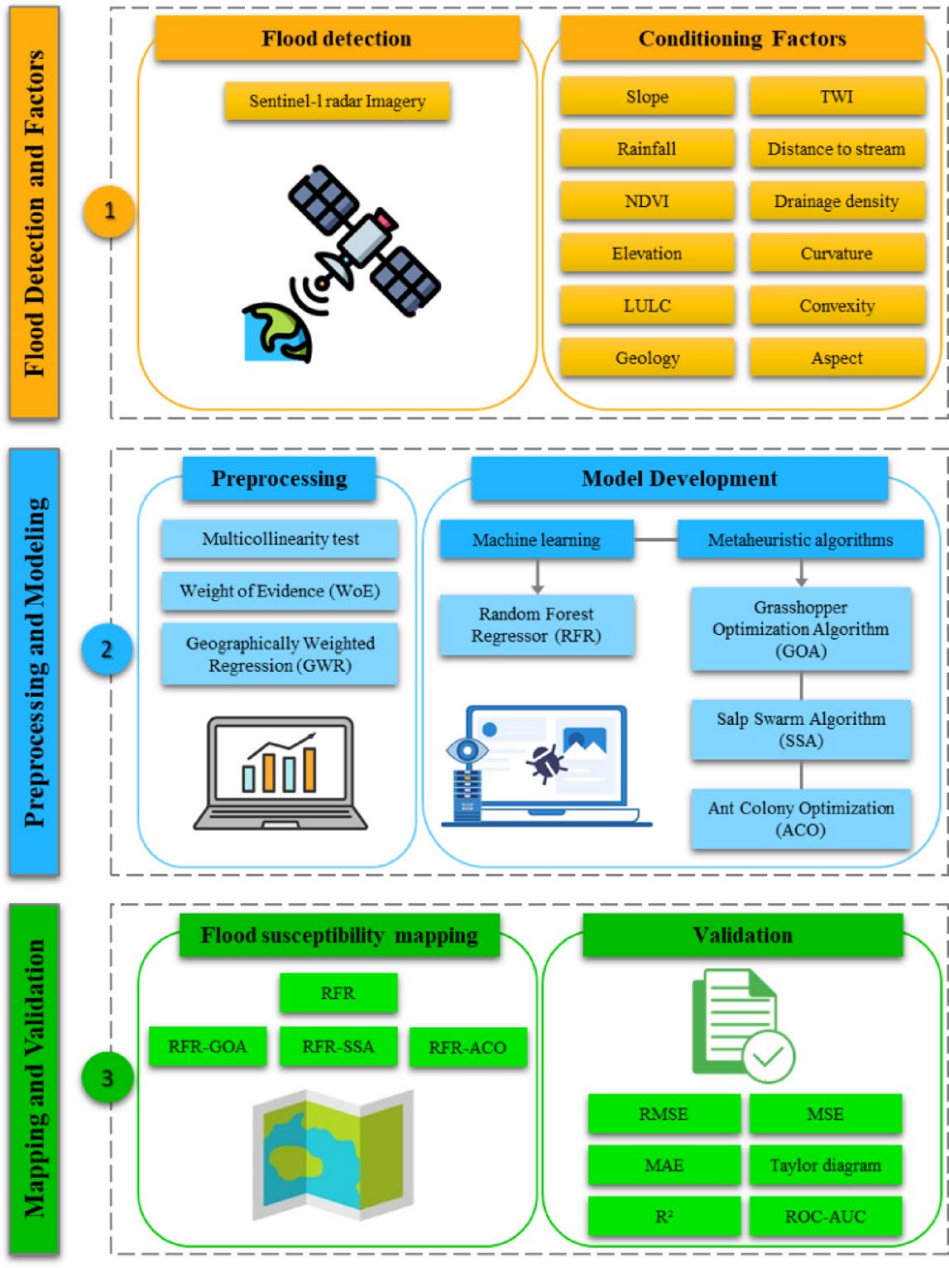
Methodological framework of the study.

This research employs a structured approach (as shown in Fig. 2) that combines data gathering, model building, and assessment of results to develop reliableflood susceptibility maps. Initially, various data sources were gathered and aligned, such as Sentinel-1 satellite radar images, weather data, water flow measurements, and terrain details. Using this information, regions impacted by floods during two past incidents (September 25, 2017, and August 25, 2019) were identified, and twelve key factors influencing floods were chosen, refined, and normalized for subsequent examination. In the subsequent modeling stage, the significance of these factors was analyzed, and forecasting models were developed. The Weight of Evidence (WoE) technique was applied to allocate statistical values to different factor categories. A check for multicollinearity was performed to evaluate relationships between variables, and Geographically Weighted Regression (GWR) was utilized to examine their spatial variablity. The core model relied on the Random Forest Regression (RFR) method, which was improved using three nature-inspired optimization techniques: Grasshopper Optimization Algorithm (GOA), Salp Swarm Algorithm (SSA), and Ant Colony Optimization (ACO). Ultimately, FSMs were produced with RFR alone and its optimized versions (RFR-GOA, RFR-SSA, and RFR-ACO). The effectiveness of these models was thoroughly tested via standard statistical metrics, confirming their strength and the trustworthiness of the generated maps.

### Flood detection

**Fig. 3:**
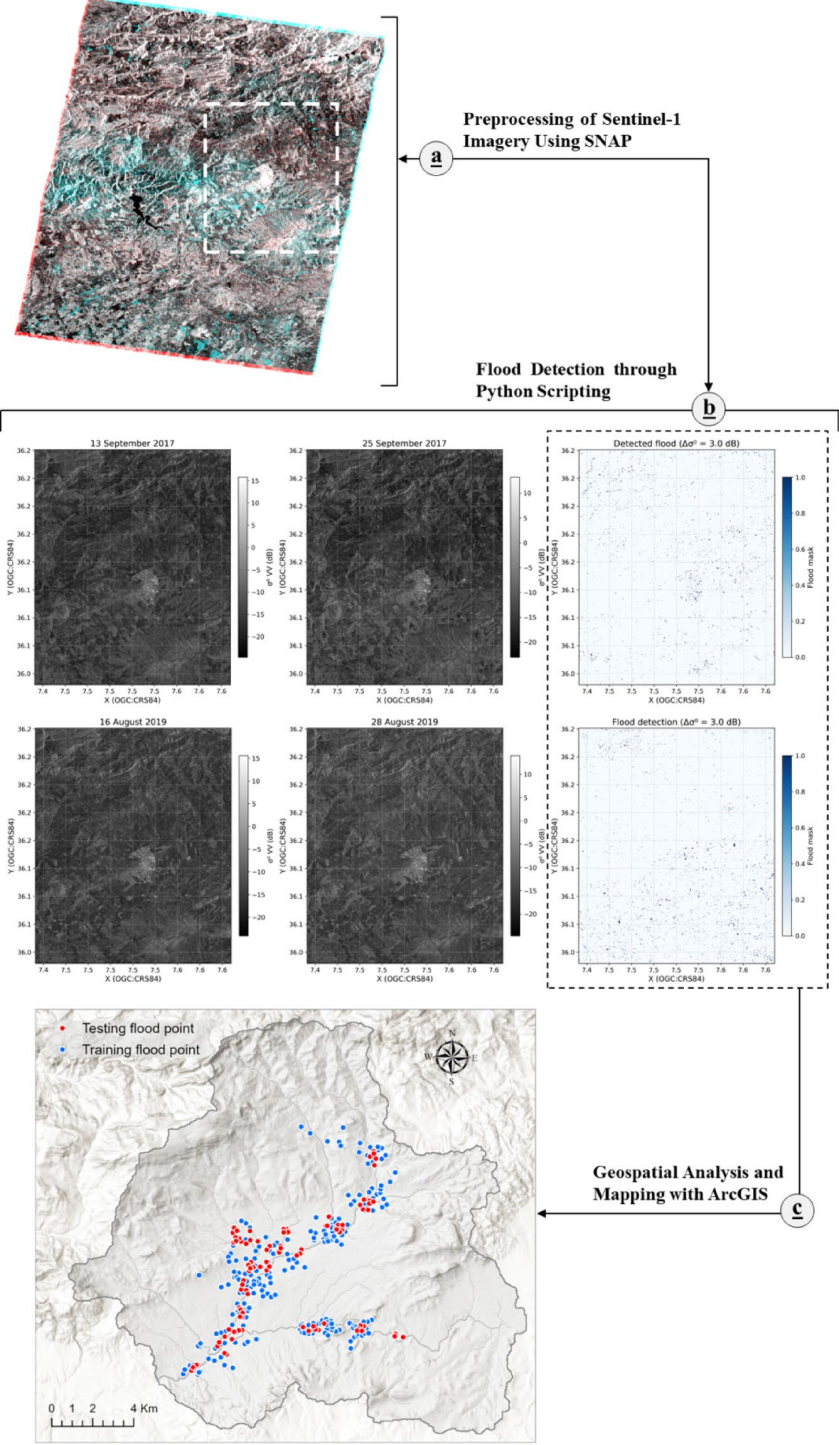
Integrated approach for flood detection.

Flood occurrences were identified using Sentinel-1 radar imagery through a three-stage procedure (Fig. 3). Initially, SAR data from before and after the events underwent preprocessing in the Sentinel Application Platform (SNAP), which involved eliminating thermal noise, performing radiometric calibration, applying speckle reduction via the Refined Lee filter (with a 3 × 3 kernel), and conducting terrain correction for geometric alignment and georeferencing (Fig. 3a). These processed images were subsequently aligned through co-registration and transformed into decibel units to allow for consistent comparison over time. A custom Python program was then used to detect flooding. The region of focus was clipped via a bounding box (BBOX), and the images from pre- and post-event periods were reprojected onto a uniform grid. A difference in decibel values was calculated to emphasize the reduction in backscatter typical of flooded zones. By imposing a constant threshold of −3.0 dB, a binary classification mask was created (Fig. 3b) and saved as a GeoTIFF for use in GIS applications. This method aligns with contemporary techniques relying on backscatter deviations, as shown in works by Amitrano et al^75^. and Sai Bhageerath et al^76^.

Next, the GeoTIFF was examined in ArcGIS, where inundated zones were converted to vector format, excluding permanent water features, and the outputs were transformed into polygons before being sampled as points for modeling purposes (Fig. 3c). Indeed, flood polygons extracted from Sentinel-1 imagery were converted into point features by computing the geometric centroid of each polygon. The centroid was selected as a representative location to reduce boundary-related uncertainties, avoid spatial redundancy, and ensure a one-to-one correspondence between flood occurrences and conditioning factor values extracted from raster datasets. This strategy is consistent with several recent flood susceptibility and hazard studies^77,78^ and^79^ among others) that use point-based inventories derived from SAR‑mapped flood extents, where flooded pixels are treated as discrete presence samples for machine learning models.

The aforementioned process was implemented for two distinct flood incidents: the event on September 25, 2017 (using images from September 13 and 25, 2017) and the one on August 25, 2019 (using images from August 16 and 28, 2019), promoting uniformity in the outcomes. The derived points were divided using a Holdout strategy as per Riazi et al^80^., allocating 70% for model training (n = 222) and 30% for validation (n = 95). For dataset equilibrium, an equal number of flooded (assigned value 1) and non-flooded (assigned value 0) points were incorporated, following current best practices in machine learning for flood delineation^81,82^.

### Conditioning factors

In this research, the choice of factors influencing flash floods was madeby a thorough review of existing studies, discussions with experts, and based on the specific physical, hydrological, weather-related, and human-induced features of the study region. Considering the standard methods used in evaluations of flood vulnerability^83,84^, a total of twelve variables were incorporated: slope, precipitation, NDVI, altitude, land use/land cover (LULC), geological composition, topographic wetness index (TWI), proximity to watercourses, drainage density, curvature, convexity, and aspect (Table 1). These elements offer a solid depiction of landscape variations, ground features, and water flow mechanisms, thereby establishing a strong foundation for modeling and mapping flash flood risks. Topographic parameters were derived from the Shuttle Radar Topography Mission (SRTM) Digital Elevation Model (DEM) with a spatial resolution of 30 m, obtained from the USGS Earth Explorer platform. Slope, aspect, curvature, and convexity were calculated using standard terrain analysis tools in ArcGIS.

**Table 1:** Dataset used in the present study.

Factor	Data source	Data type	Spatial resolution
Elevation	SRTM DEM (USGS Earth Explorer)	Raster	30 m
Slope	Derivedfrom SRTM DEM	Raster	30 m
Aspect	Derivedfrom SRTM DEM	Raster	30 m
Curvature	Derivedfrom SRTM DEM	Raster	30 m
Convexity	Derivedfrom SRTM DEM	Raster	30 m
TWI	Derivedfrom SRTM DEM	Raster	30 m
Distance to streams	Derived from DEM-based drainage network	Raster	30 m
Drainage density	Derived from DEM-based drainage network	Raster	30 m
Rainfall	Four meteorological stations (National Meteorological Office—ONM, Algeria)	Point/Raster (interpolated)	30 m
NDVI	Sentinel-2A imagery (Copernicus Open Access Hub)	Raster	10 m (resampled to 30 m)
LULC	Sentinel-2A imagery (Copernicus Open Access Hub)	Vector/Raster	10 m (resampled to 30 m)
Geology	Geological map of Algeria (ORGM)	Vector	1:50,000

### Slope

**Fig. 4:**
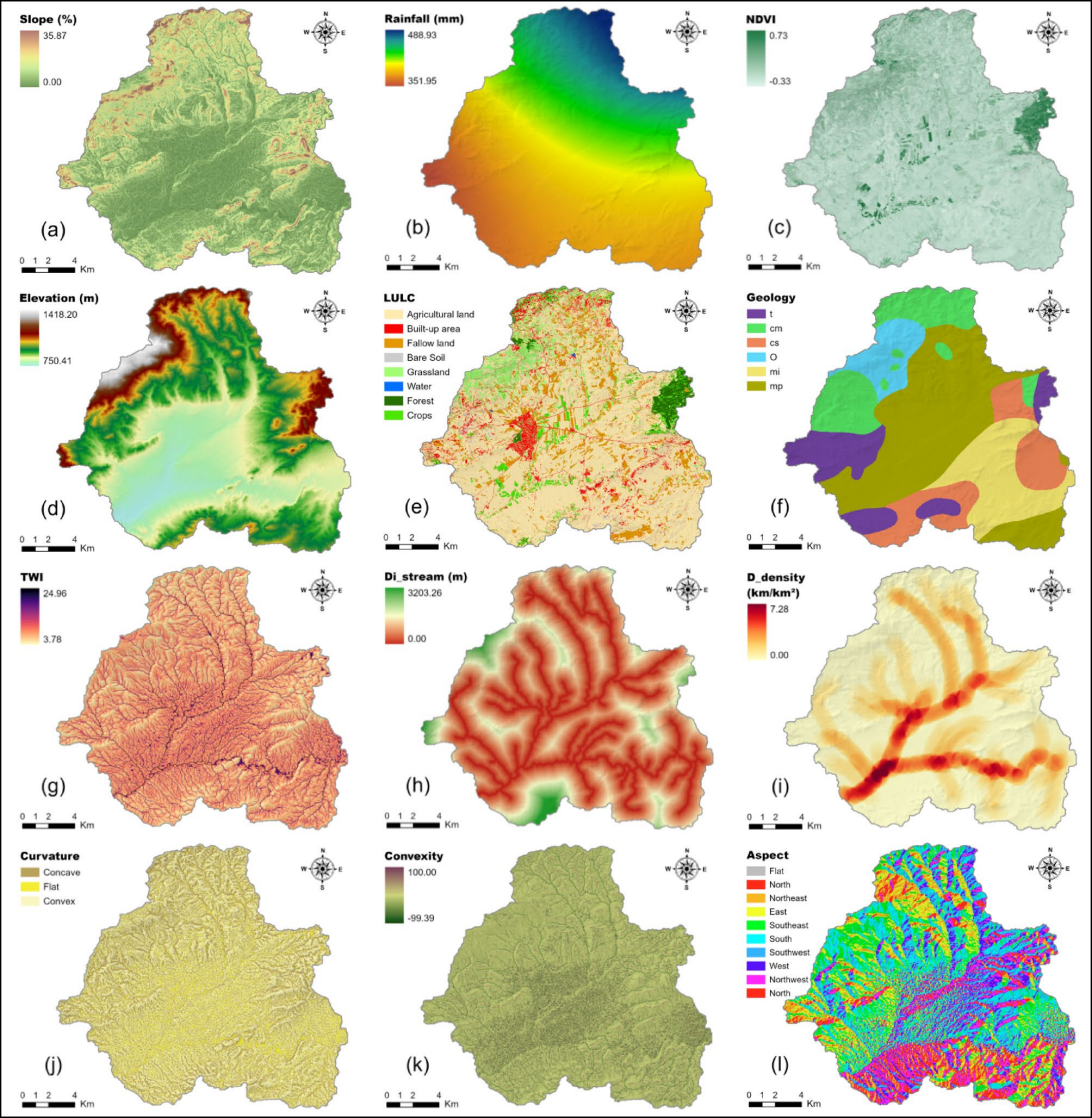
Different flood conditioning factors: **a** Slope, **b** Rainfall, **c** NDVI, **d** Elevation, **e** LULC, **f** Geology, **g** TWI, **h** Distance to stream, **i** Drainage density, **j** Curvature, **k** Convexity, **l** Aspect.

Slope measures the degree of elevation variation over a given horizontal span, obtained from a digital elevation model (DEM). Within flood simulations, it plays a key role in determining the speed of surface water flow and the time for water to accumulate: flatter areas tend to decelerate drainage, promote soil absorption, and potentially heighten flood exposure locally, while sharper inclines speed up water movement and shorten retention periods, often lowering the chances of sudden flooding in those spots^85,86^. For this dataset, slope measurements span from 0 to 35.9% (Fig. 4a).

### Rainfall

Rainfall serves as the main weather driver for flash floods, with the strength and length of storms dictating water overflow and maximum flow rates^87,88^. Here, yearly average precipitation figures were obtained from four monitoring stations, operated by the Algerian National Office of Meteorology (ONM), were spatially mapped via the inverse distance weighting (IDW) technique within ArcGIS to generate a continuous rainfall raster at 30 m spatial resolution. The figures vary between 352.0 and 489.0 mm throughout the catchment area (Fig. 4b).

### NDVI

The Normalized Difference Vegetation Index (NDVI) is a common remote sensing metric for evaluating plant coverage and health, determined by:

1 $$NDVI = \frac{NIR - RED}{{NIR + RED}}$$

where ${\mathrm{NIR}}$ represents near-infrared reflectance (Sentinel-2A Band 3) and $\mathrm{RED}$ represents red reflectance (Sentinel-2A Band 4). High positive values correspond to dense and healthy vegetation, which enhances infiltration and reduces surface runoff, while values near zero or negative indicate bare soil, urban surfaces, or water bodies. In flood susceptibility studies, low vegetation cover is typically associated with increased runoff potential and higher risk^68–71,89^. In this study, Sentinel-2A imagery (10 m spatial resolution) was used to compute the Normalized Difference Vegetation Index (NDVI) and to produce the Land Use/Land Cover (LULC) map. To ensure spatial consistency with other conditioning factors, all Sentinel-2A-derived products were resampled to a common resolution of 30 × 30 m. Obtained NDVI values range from −0.33 to + 0.73 (Fig. 4c).

### Elevation

Elevation, obtained from the digital elevation model (DEM), affects water flow routes by establishing the landscape’s incline and overall height differences. Regions at lower altitudes typically gather water from higher grounds, making them more prone to inundation^90,91^. Within the examined zone, heights vary between 750.41 and 1,418.20 m (Fig. 4d).

### Land use/land cover (LULC)

The patterns of land use and cover dictate the way ground surfaces react to precipitation, by adjusting factors like water capture, soil absorption, terrain friction, and moisture evaporation. Plant-covered zones usually minimize water overflow, while sealed or exposed grounds encourage quicker drainage^92,93^. For this analysis, classification of a Sentinel-2A satellite image revealed eight categories: Urban development, Exposed earth, Farm fields, Cultivated crops, Idle farmland, Pasture, Woodland, and Aquatic features (Fig. 4e). Locations featuring sparse plants or extensive sealed surfaces are viewed as having greater vulnerability to sudden floods.

### Geology

Geologic formations impact underground attributes including permeability, water seepage rates, and aquifer linkages. Different rock types differ in terms of pore spaces, crack systems, or layered arrangements, which regulate how water is divided between overland flow and below-ground movement. In the studied basin, six rock types (Fig. 4f) were pinpointed using the 1:50,000 scale geologic chart from ORGM:

1. t Marine/Lagoonal Triassic
2. o Marine Oligocene (+ local Upper Eocene)
3. cm Marine/Lagoonal Middle Cretaceous
4. cs Marine Upper Cretaceous
5. mi Lower Marine Miocene (Burdigalian)
6. mpPontian/Messinian.

These heterogeneous lithologies result in spatial variations in groundwater recharge and runoff pathways, influencing local flood susceptibility.

### Topographic wetness index (TWI)

The TWI is a morphometric measure combining upslope contributing area and local slope to estimate soil moisture accumulation potential:

2 $$TWI = ln\left( {\frac{{A_{s} }}{tan\beta }} \right)$$

where $A_{s}$ is the specific contributing area (m^2^ per unit contour width) and ${\upbeta }$ is the local slope angle in radians. High TWI values generally indicate terrain prone to saturation or water accumulation, thereby identifying flood-prone areas^94^. In this study, the Topographic Wetness Index (TWI) was computed based on upslope contributing area and local slope gradient derived from the DEM. TWI values range from 3.78 to 24.96 (Fig. 4g).

### Distance to streams

Proximity to the closest waterway acts as a vital geographic factor in flood simulations, given that channels aggregate and transport excess water. Grid cells situated near the stream system face higher risks from overbank flows or embankment collapses, thereby increasing their flood vulnerability^32,33,95^. In the analyzed region, distances to rivers extend from 0 to 3,203 m (Fig. 4h).

### Drainage density

Drainage density represents the overall length of watercourses divided by the catchment area (km/km^2^). This indicator reveals a watershed’s capability to release surface water: elevated densities often signal intensified water collection and diminished soil penetration, which can encourage rapid flood development^68–71,96^. Within this work, the drainage network was extracted using flow direction and flow accumulation algorithms, from which drainage density and distance to streams were calculated. Drainage density values vary between 0 and 7.28 km/km^2^ (Fig. 4i).

### Curvature

Curvature evaluates the second-order change in the land surface, illustrating variations in incline. Based on its polarity, the ground may appear outwardly curved (positive), inwardly curved (negative), or level (neutral). Outwardly curved sections typically hasten water movement and curb pooling, whereas inwardly curved areas facilitate stream merging, storage, or seepage^97^. Curvature values stretched from −1.88 (intensely concave) to + 2.44 (intensely convex) (Fig. 4j).

### Convexity

Convexity, alternatively termed the convergence index, serves as a holistic gauge of terrain morphology, differentiating crests (marked by strong positive values) from troughs (indicated by negative values or convergence). Areas with pronounced convexity enhance drainage and restrict water gathering, while converging configurations direct flows and support moisture holding. In the present investigation, convexity spans from −99.39 to + 100 (Fig. 4k), spanning a broad range of shapes. These metrics are routinely employed in analyses of earth forms and water hazard assessments^98^.

### Aspect

Aspect denotes the directional facing of slopes (0–360° from north), with 0° for north, 90° for east, 180° for south, and 270° for west. In environments ranging from dry to somewhat moist, this orientation affects sunlight intake, vaporization rates, and earth hydration, which indirectly impacts overflow and percolation. For example, inclines oriented toward the north or east (with reduced solar contact in peak times) generally preserve more humidity and might display elevated tendencies for surplus runoff or buildup^99,100^. In this analysis, aspect was computed from the DEM via ArcGIS (Fig. 4l).

### Multicollinearity analysis

Multicollinearity describes the presence of substantial linear correlations among two or more explanatory variables in a regression analysis. While it does not inherently compromise the model’s overall predictive accuracy, it does amplify the variance associated with coefficient estimates and obscures the distinct roles played by individual predictors, ultimately eroding the dependability of statistical conclusions^85,101,102^. Commonly used diagnostics for identifying multicollinearity include the Tolerance (TOL) and Variance Inflation Factor (VIF). The relevant equations are outlined as follows:

3 $$TOL = 1 - R_{j}^{2}$$

4 $$VIF = \frac{1}{TOL}$$

where $R_{j}^{2}$ is the coefficient of determination obtained by regressing the $j^{th}$ independent variable against all other predictors. In general, VIF > 10 and TOL < 0.1 are considered strong indicators of severe multicollinearity^46,47^. In the present study on flash flood susceptibility, a multicollinearity test was carried out prior to model implementation to ensure statistical reliability and to minimize redundancy among conditioning factors.

### Weight of evidence (WoE) method

The Weight of Evidence (WoE) technique serves as a Bayesian statistical framework, grounded in Bayes’ theorem (Bonham-Carter, 1994) ^145^. It has seen widespread use in mapping susceptibility to natural hazards, with notable applications in areas such as flash flood prediction, landslide assessment, erosion dynamics, and groundwater potential evaluation^103–107^. Essentially, this method assesses the spatial connections between an event’s occurrence and a suite of influencing factors by assigning statistical weights to the various categories within those factors. These weights capture the degree to which the presence or absence of a specific category heightens or lowers the probability of the event unfolding.

For a given class $\left( {B_{i} } \right)$, the positive weight is defined as (Eq. 5):

5 $$W^{ + } = ln\left[ {\frac{{P\left( {B_{i} {|}A} \right)}}{{P\left( {B_{i} {|}\overline{A}} \right)}}} \right]$$

and the negative weight is calculated as (Eq. 6):

6 $$W^{ - } = ln\left[ {\frac{{P\left( {\overline{B}_{i} {|}A} \right)}}{{P\left( {\overline{B}_{i} {|}\overline{A}} \right)}}} \right]$$

where $A$ et $\overline{A}$ denote the presence and absence of flash floods, respectively, and $B_{i}$, $\overline{B}_{i}$ the presence or absence of the conditioning factor. The final weight is obtained as the difference between the two values (Eq. 7):

7 $$W_{fin} = W^{ + } - W^{ - }$$

A positive $W_{fin}$ indicates that the factor class contributes to the occurrence of flash floods, whereas a negative value reflects a reducing effect. When implemented in a GIS environment with raster data, the WoE method provides a transparent and reproducible framework for susceptibility mapping, though it requires the assumption of conditional independence among the input factors^108,109^. This study applies the Weight of Evidence (WoE) method to rank the relative importance of conditioning factors and to identify the classes that encompass the largest number of flash-flood pixels.

### Geographically weighted regression (GWR) method

Geographically Weighted Regression (GWR) is a spatial statistical method developed to account for spatial non-stationarity in regression analysis^110,111^. Unlike global regression models, which assume uniform relationships across space, GWR allows regression coefficients to vary locally, thus capturing geographic heterogeneity. The model is expressed as:

8 $$y_{i} = \beta_{0} \left( {u_{i} ,v_{i} } \right) + \mathop \sum \limits_{k = 1}^{p} \beta_{k} \left( {u_{i} ,v_{i} } \right)x_{ik} + \varepsilon_{i}$$

where $y_{i}$ is the dependent variable at location $i;\left( {u_{i} ,v_{i} } \right)$ are the coordinates; $\beta_{k} \left( {u_{i} ,v_{i} } \right)$ are location-specific coefficients; $\varepsilon_{i}$ and is the error term^112^. Spatial weighting is introduced through a kernel function, commonly Gaussian:

9 $$W_{ij} = exp\left( { - \frac{{d_{ij}^{2} }}{{b^{2} }}} \right)$$

with $d_{ij}$ the distance between locations $i$ and $j$, and $b$ the bandwidth controlling spatial influence (Nakaya , 2015) ^146^. By calibrating coefficients locally, GWR provides a more realistic representation of environmental processes, making it widely applied in hydrology and flood susceptibility studies^87,113^. In this study, the GWR method was applied with the aim of assessing spatial variations in the influence of topographic, hydrological, and climatic factors on flash flood susceptibility.

### Random forest regressor model

The Random Forest Regressor (RFR) is an ensemble learning algorithm introduced by Leo Breiman^53^ that aggregates multiple decision trees to enhance prediction accuracy and reduce overfitting. It is based on the *bagging* (bootstrap aggregating) principle, where each tree is trained on a random bootstrap sample of the training dataset and on a random subset of features^114,115^. Given a regressiondataset

10 $$D = \left\{ {\left( {x_{i} ,y_{i} } \right)} \right\}_{i = 1}^{N}$$

where $x_{i} \in { mathbb{R}}^{p}$ is the feature vector and ${\mathrm{y}}_{{\mathrm{i}}} \in { mathbb{R}}$ the target variable, each decision tree $h_{b} \left( x \right)$ is trained on a bootstrap sample $D_{b}$ drawn from $D$. At each node, only $m < p$ randomly selected features are considered for splitting to reduce correlation among trees. The Random Forest prediction for a new input $x$ is the average of all individual tree predictions:

11 $$\hat{f}_{RF} \left( x \right) = \frac{1}{B}\mathop \sum \limits_{b = 1}^{B} h_{b} \left( x \right)$$

where $B$ is the total number of trees.

Due to its variance-reduction property and robustness to noise and nonlinearity, the RFR has shown strong performance in flash flood susceptibility modeling, effectively capturing complex spatial and hydrological relationships^32,33,98^

### Grasshopper optimization algorithm (GOA)

The Grasshopper Optimization Algorithm (GOA) represents a population-based metaheuristic approach, drawing inspiration from the swarming dynamics observed in grasshopper populations^72^. It effectively captures the interplay between exploratory behaviorsfacilitating broad, global searchesand exploitative strategies, which focus on precise local refinements, by incorporating three core components: social interactions among individuals, gravitational forces, and wind-driven advection. This framework has demonstrated notable efficacy in tackling nonlinear and multimodal optimization challenges, rendering it particularly apt for applications in spatial forecasting, including the assessment of flash flood vulnerability^116,117^. Mathematically, the position of the ${\boldsymbol{i}}^{{{\boldsymbol{th}}}}$ grasshopper in the search space is expressed as:

12 $$X_{i} = S_{i} + G_{i} + A_{i}$$

where $S_{i}$ represents the social interaction force among individuals, $G_{i}$ the gravitational pull, and $A_{i}$ the wind advection component. The social interaction component is modeled as:

13 $$S_{i} = \mathop \sum \limits_{{\begin{array}{*{20}c} {j = 1} \\ {j \ne 1} \\ \end{array} }}^{N} s\left( {d_{ij} } \right)\hat{d}_{ij}$$

where $d_{ij}$ is the Euclidean distance between individuals $i$ and $j$, and $\hat{d}_{ij}$ is the unit direction vector from $j$ to $i$. The interaction intensity function $s\left( {d_{ij} } \right)$ regulates attraction and repulsion according to:

14 $$s\left( r \right) = fe^{ - r/\ell } - e^{ - r}$$

where $f$ is the attraction strength and $\ell$ is the interaction length scale.

This formulation enables the Grasshopper Optimization Algorithm (GOA) to achieve a dynamic equilibrium between diversification encompassing broad, global exploration and intensification, which emphasizes targeted local searches. Consequently, it facilitates more effective navigation of intricate optimization terrains^118,119^. Nevertheless, GOA is prone to drawbacks such as early convergence or diminished diversity within the population, especially in scenarios involving high-dimensional spaces or highly irregular fitness landscapes^120^. To mitigate these challenges, a number of contemporary investigations have put forward hybrid and adaptive modifications, including refined versions of GOA incorporating random weighting or chaotic mutations, which aim to accelerate convergence while evading entrapment in local optima^121,122^.

### Salp swarm algorithm (SSA)

The Salp Swarm Algorithm (SSA) constitutes a swarm-based metaheuristic technique, modeled after the sociable and linear foraging dynamics of salps^73^. In the context of optimization, SSA replicates this synchronized locomotion to strike a balance between exploratory endeavors (wide-ranging probes for auspicious domains) and exploitative refinements (intensive adjustments proximate to superior outcomes), thereby rendering it especially appropriate for tackling non-linear and geographically multifaceted issues, such as the delineation of flash flood scenarios^123^. Within SSA, the ensemble is segmented into leaders and followers, wherein the vanguard salps steer the collective toward the nutrient source (symbolizing the ideal solution), while the subsequent members recalibrate their placements in relation to their immediate predecessors in the formation. The position of the $j^{th}$ dimension of the leader salp is updated as follows:

15 $$x_{j}^{1} = \left\{ {\begin{array}{*{20}c} {F_{j} + c_{1} \left( {\left( {ub_{j} - lb_{j} } \right)c_{2} + lb_{j} } \right),\quad if\;c_{3} \ge 0} \\ {F_{j} + c_{1} \left( {\left( {ub_{j} - lb_{j} } \right)c_{2} + lb_{j} } \right),\quad if\;c_{3} < 0} \\ \end{array} } \right.$$

where $F_{j}$ denotes the position of the food source in the $j^{th}$ dimension, $ub_{j}$ and $lb_{j}$ are the upper and lower bounds of the search space, and $c_{1} ,{ }c_{2} { } \in { }\left[ {0,{ }1} \right]$ are random numbers controlling stochastic motion. The control coefficient $c_{1}$ decreases nonlinearly with iterations to balance exploration and exploitation:

16 $$c_{1} = 2e^{{ - \left( \frac{4t}{T} \right)^{2} }}$$

where $t$ and $T$ denote the current and total number of iterations, respectively. The followers adjust their positions by averaging their current location with that of the salp directly ahead:

17 $$x_{j}^{i} = \frac{1}{2}\left( {x_{j}^{i} + x_{j}^{i - 1} } \right),\quad i \ge 2$$

This mechanism creates a smooth convergence toward the optimal region while maintaining the swarm’s diversity. Despite its simplicity, SSA has demonstrated robust performance in environmental modeling, parameter tuning, and feature selection tasks^124,125^.

### Ant colony optimization (ACO) algorithm

The Ant Colony Optimization (ACO) method constitutes a swarm-intelligence metaheuristic, drawing inspiration from the indirect, pheromone-mediated foraging strategies exhibited by natural ant colonies^74^. In domains like flash flood vulnerability evaluation, ACO facilitates the adept traversal of intricate, multidimensional optimization landscapes, yielding near-optimal parameter settings or geospatial configurations through the synergistic actions of agents and adaptive pheromone-based feedback^126,127^.

In ACO, each artificial ant constructs a candidate solution by traversing the problem graph, where the probability of moving from node ${\mathrm{i}}$ to node ${\mathrm{j}}$ depends on both the pheromone intensity $\left( {\tau_{ij} } \right)$ and a heuristic factor $\left( {\eta_{ij} } \right)$, as expressed by:

18 $$P_{ij} = \frac{{\left( {\tau_{ij} } \right)^{\alpha } \left( {\eta_{ij} } \right)^{\beta } }}{{\mathop \sum \nolimits_{k \in allowed} \left( {\tau_{ik} } \right)^{\alpha } \left( {\eta_{ik} } \right)^{\beta } }}$$

where $\alpha$ and $\beta$ control the relative influence of pheromone concentration and heuristic desirability, respectively^128^.

Following solution construction, pheromone trails are updated to reinforce successful paths and enable adaptive learning through positive feedback, while allowing pheromone evaporation to avoid premature convergence. The pheromone update rule is given by:

19 $$\tau_{ij}^{\left( n \right)} = \left( {1 - \rho } \right)\tau_{ij}^{\left( s \right)} + \Delta \tau_{ij}$$

where $\rho$ is the evaporation rate $(0 < \rho < 1)$, $\tau_{ij}^{\left( s \right)}$ denotes the previous pheromone value, and $\Delta \tau_{ij}$ represents the pheromone deposited by the ants based on solution quality^129^.

### Metaheuristic optimization of the RFR model

Enhancing the performance of machine learning algorithms represents a pivotal phase in bolstering the accuracy, robustness, and extrapolative potential of models designed for flash flood susceptibility assessment. Within this investigation, metaheuristic optimization strategies were seamlessly incorporated into the Random Forest Regression (RFR) architecture to facilitate automated calibration of its essential hyperparameters, thereby mitigating the inherent prejudices of manual tuning^59,130^. Three biologically motivated optimization methods were utilized: the Grasshopper Optimization Algorithm (GOA), the Salp Swarm Algorithm (SSA), and the Ant Colony Optimization (ACO). These swarm-intelligence paradigms emulate the collaborative wisdom and dynamic adaptations observed in living systems, enabling proficient navigation through intricate, high-dimensional, and non-linear optimization landscapes^72,119,131^. The fine-tuning efforts concentrated on critical RFR parameters, such as the ensemble size (n_estimators), the upper limit on tree depth (max_depth), and the subset of features evaluated per node split (max_features). The overarching aim was to diminish the Mean Squared Error (MSE), ultimately elevating the model’s forecasting efficacy.

### Model validation

To safeguard the dependability, resilience, and extrapolative prowess of the formulated flood susceptibility models, a suite of statistical and visual evaluative measures was utilized, including the root mean squared error (RMSE), mean absolute error (MAE), mean squared error (MSE), coefficient of determination (R^2^), and Taylor diagrams. These metrics were derived from the continuous projected flood susceptibility values (spanning 0 to 1) juxtaposed against the binary empirical flood inventory records (where flood occurrence = 1 and non-occurrence = 0), applied across 317 distinct validation sites. This multifaceted appraisal methodology facilitates a precise appraisal of both the forecasting accuracy and the congruence between simulated and empirical flood distributions^46,47,132^..

### RMSE, MAE, and MSE

The RMSE, MAE, and MSE are among the most widely used and interpretable statistical indicators for model validation, particularly within geospatial and hydrological modeling contexts^66,133^. These metrics quantify the magnitude of prediction errors between observed and estimated values as follows:

20 $$RMSE = \sqrt {\frac{1}{n}\mathop \sum \limits_{i = 1}^{n} \left( {X_{i} - \hat{X}_{i} } \right)^{2} }$$

21 $$MAE = \frac{1}{n}\mathop \sum \limits_{i = 1}^{n} \left| {X_{i} - \hat{X}_{i} } \right|$$

22 $$MSE = \frac{1}{n}\mathop \sum \limits_{i = 1}^{n} \left( {X_{i} - \hat{X}_{i} } \right)^{2}$$

where $n$ is the total number of samples, $X_{i}$ represents the observed flood occurrence, and $\hat{X}_{i}$ denotes the model-predicted flood susceptibility. Lower values of RMSE, MAE, and MSE indicate a better model fit and higher predictive accuracy. These indicators are particularly useful for comparing machine learning and metaheuristic optimization models^50^.

### Coefficient of determination $\left( {{\boldsymbol{R}}^{{\mathbf{2}}} } \right)$

The Coefficient of Determination $\left( {R^{2} } \right)$ expresses the proportion of variance in the observed data explained by the model and is calculated as:

23 $$R^{2} = 1 - \frac{{\mathop \sum \nolimits_{i = 1}^{n} \left( {X_{i} - \hat{X}_{i} } \right)^{2} }}{{\mathop \sum \nolimits_{i = 1}^{n} \left( {X_{i} - \overline{X}} \right)^{2} }}$$

where $\overline{X}$ is the mean of observed values. An $R^{2}$ value approaching 1 indicates a strong linear correlation and highexplanatory power of the model^134^. In flood susceptibility modeling, $R^{2}$ complements error based metrics by highlighting the overall strength of the predictive relationship rather than the absolute magnitude of residuals.

### Taylor diagram

The Taylor Diagram provides a concise and powerful graphical summary of model performance by simultaneously displaying the correlation coefficient, standard deviation, and centered RMSE^135^. It facilitates multi-metric comparisons among various models, such as: RFR, RFR-GOA, RFR-SSA, and RFR-ACO relative to reference observations. Models positioned closer to the reference point exhibit higher correlation and lower error, indicating superior predictive capability^31,136^.

### ROC curve

The Receiver Operating Characteristic (ROC) curve illustrates the trade-off between the True Positive Rate (TPR) and the False Positive Rate (FPR) as the classification threshold varies^137,138^. The TPR represents the proportion of flood-prone locations correctly identified as positive, whereas the FPR denotes the proportion of non-flood locations incorrectly classified as flood-prone^68–71^. The Area Under the Curve (AUC) summarizes the ROC performance into a single scalar value: an AUC of 1.0 indicates perfect discrimination, while an AUC of 0.5 corresponds to random classification performance^139^. Formally, the AUC can be expressed as:

24 $$AUC = \frac{1}{{\left| P \right|{ }.\left| N \right|}}\mathop \sum \limits_{i \in P} \mathop \sum \limits_{j \in N} 1\left( {s_{i}> s_{j} } \right)$$

where $P$ and $N$ denote the sets of flood and non-flood samples, respectively, and $s_{i}$ and $s_{j}$​ are their predicted scores.

## Result

### Results of multicollinearity analysis

The findings outlined in Table 2 reveal that all explanatory variables possess Variance Inflation Factor (VIF) scores beneath the established threshold of 10, signifying a lack of pronounced multicollinearity. Notably, elevation and precipitation exhibit the most elevated VIF figures (6.74 and 6.07, respectively), which suggest a reasonable degree of interrelation with select other factors. Conversely, aspect yields the minimal VIF (1.10). The tolerance metrics, uniformly surpassing 0.1, additionally corroborate the robustness and comparative autonomy of these variables. Consequently, the full suite of variables was incorporated into the modeling framework, with each offering a unique and synergistic insight into the mechanisms underpinning flash flood initiation.

**Table 2:** Multicollinearity analysis results.

Variable	VIF	Tolérance
Slope	2.43727	0.41030
TWI	2.06240	0.48487
Elevation	6.74424	0.14828
Rainfall	6.07081	0.16472
NDVI	1.99778	0.50056
D_stream	3.18204	0.31426
D_density	2.65558	0.37657
Curvature	1.47837	0.67642
Convexity	1.81889	0.54979
Aspect	1.10617	0.90402
LULC	2.12393	0.47083
Geology	1.46640	0.68194

### Result of weight of evidence (WOE) method

The outcomes from the Weight of Evidence (WOE) analysis (Table 3) elucidate the primary determinants of flash-flood events within the study region. In terms of slope, the 0–2% category exhibits the most pronounced correlation with flash floods (58 pixels), affirming the heightened susceptibility of gently sloping terrains to water accumulation. For the Topographic Wetness Index (TWI), the 7–10 range emerges as the most influential (59 pixels), indicating that areas of intermediate moisture levels are particularly conducive to flash-flood aggregation. Elevation data reveal that lower altitudes, specifically between 750 and 820 m, are most prone to flooding (75 pixels). Regarding precipitation, the class below 380 mm registers the greatest incidence of flash-flood pixels (62), implying that such events are not solely driven by intense rainfall but are modulated by site-specific geomorphic and hydrologic attributes. Vegetation density, as measured by the Normalized Difference Vegetation Index (NDVI), shows dominance in the 0.10–0.30 interval (68 pixels), highlighting the exposure of sparsely vegetated zones. River proximity exerts a substantial effect, with the 0–200 m zone capturing 51 flash-flood pixels. Drainage density is likewise pivotal, as the 3.0–5.0 km/km^2^ category demonstrates the strongest association (45 pixels). Curvature assessments indicate that planar surfaces are most linked to flooding (53 pixels). The convergence index identifies the −40 to −10 range as particularly critical (30 pixels). Slope aspect reveals greater vulnerability on southwest- and west-oriented facets (18 pixels each). Land use patterns underscore agricultural lands as the most at-risk (51 pixels), trailed by urban developments (17 pixels), whereas forested areas and water features show no such linkage. Lastly, lithological factors pinpoint the Pontian/Messinian formation as the predominant contributor (76 pixels), underscoring the significant role of underlying geology in governing flash-flood dynamics.

**Table 3:** Weight of evidence (WOE) values and spatial distribution of flash-flood pixels by factor classes.

Factors	Interval	Score	W +	W−	W_fin_	Flash-Flood pixels
Slope (%)	0 to 2	5	− 0.490099	− 0.510826	0.020726	58
	2 to 5	4	− 1.174736	− 1.067636	− 0.107100	29
	5 to 10	3	− 2.544223	− 2.962580	0.418357	7
	10 to 20	2	− 4.153661	− 4.488636	0.334975	1
	> 20	1	− 5.252273	− 6.098074	0.845801	0
TopographicWetness Index (TWI)	< 7	1	− 1.588712	− 1.353142	− 0.235570	19
	7 to 10	2	− 0.473150	− 0.481303	0.008153	59
	10 to 13	3	− 2.116779	− 2.434513	0.317733	11
	13 to 16	4	− 2.854378	− 3.153635	0.299257	5
	> 16	5	− 4.153661	− 6.098074	1.944413	1
Elevation (m)	750 to 820	5	− 0.234994	− 0.214752	− 0.020242	75
	820 to 900	4	− 1.641356	− 1.703625	0.062270	18
	900 to 1000	3	− 3.642836	− 4.152164	0.509329	2
	1000 to 1200	2	− 5.252273	− 6.098074	0.845801	0
	> 1200	1	− 5.252273	− 6.098074	0.845801	0
Rainfall (mm)	< 380	1	− 0.423960	− 0.474057	0.050097	62
	380 to 410	2	− 1.047581	− 0.968176	− 0.079405	33
	> 470	5	− 5.252273	− 6.098074	0.845801	0
NDVI	− 0.33 to − 0.10	5	− 5.252273	− 4.999462	− 0.252811	0
	− 0.10 to 0.10	4	− 1.402126	− 1.482954	0.080828	23
	0.10 to 0.30	3	− 0.332293	− 0.345502	0.013209	68
	0.30 to 0.50	2	− 4.153661	− 3.533125	− 0.620536	1
	0.50 to 0.73	1	− 3.306363	− 3.264861	− 0.041502	3
Distance to Stream (m)	0 to 200	5	− 0.617544	− 0.655657	0.038112	51
	200 to 500	4	− 0.908468	− 0.888588	− 0.019880	38
	500 to 1000	3	− 2.687324	− 2.601567	− 0.085757	6
	1000 to 2000	2	− 5.252273	− 6.098074	0.845801	0
	> 2000	1	− 5.252273	− 6.098074	0.845801	0
Drainage Density (km/km^2^)	0 to 0.5	1	− 2.854378	− 2.730778	− 0.123600	5
	0.5 to 1.5	2	− 2.207751	− 2.487156	0.279405	10
	1.5 to 3.0	3	− 1.360453	− 1.222877	− 0.137576	24
	3.0 to 5.0	4	− 0.741414	− 0.866966	0.125552	45
	> 5.0	5	− 2.116779	− 1.923687	− 0.193092	11
Curvature	Concave (− 1.88 to − 0.10)	4	− 1.588712	− 1.406726	− 0.181985	19
	Flat (− 0.10 to 0.00)	3	− 0.579445	− 0.664352	0.084908	53
	Convex (0.00 to + 2.44)	2	− 1.402126	− 1.406726	0.004601	23
Convexity/Convergence Index	≤ − 40	5	− 3.055049	− 2.962580	− 0.092469	4
	− 40 to − 10	4	− 1.141400	− 1.353142	0.211743	30
	− 10 to + 10	3	− 1.174736	− 1.080794	− 0.093942	29
	+ 10 – + 40	2	− 1.402126	− 1.238262	− 0.163864	23
	> + 40	1	− 2.307834	− 2.664087	0.356253	9
Aspect	Flat	3	− 5.252273	− 6.098074	0.845801	0
	North	4	− 2.854378	− 4.152164	1.297786	5
	Northeast	4	− 2.687324	− 2.802237	0.114913	6
	East	3	− 2.307834	− 2.127782	− 0.180052	9
	Southeast	2	− 1.818286	− 1.754269	− 0.064017	15
	South	2	− 2.307834	− 1.954940	− 0.352895	9
	Southwest	2	− 1.641356	− 1.893382	0.252026	18
	West	3	− 1.641356	− 1.728626	0.087271	18
	Northwest	4	− 2.033398	− 2.127782	0.094385	12
	North	4	− 3.306363	− 2.802237	− 0.504126	3
Land Use/Land Cover	Built-up area	5	− 1.696925	− 1.632166	− 0.064759	17
	BareSoil	4	− 5.252273	− 6.098074	0.845801	0
	Agricultural land	3	− 0.617544	− 0.556811	− 0.060734	51
	Crops	3	− 2.307834	− 2.487156	0.179322	9
	Fallow land	3	− 2.207751	− 2.487156	0.279405	10
	Grassland	2	− 2.419060	− 2.730778	0.311718	8
	Forest	1	− 5.252273	− 4.999462	− 0.252811	0
	Water	**1**	− 5.252273	− 4.999462	− 0.252811	0
Geology	Marine/LagoonalTriassic	4	− 2.687324	− 2.664087	− 0.023237	6
	Marine Oligocene (+ local Upper Eocene)	4	− 5.252273	− 6.098074	0.845801	0
	Marine/Lagoonal Middle Cretaceous	3	− 5.252273	− 4.999462	− 0.252811	0
	Marine UpperCretaceous	3	− 3.055049	− 3.390024	0.334975	4
	Lower Marine Miocene (Burdigalian)	2	− 2.307834	− 2.384502	0.076668	9
	Pontian/Messinian	3	− 0.221836	− 0.214752	− 0.007084	76

### Results of the GWR analysis

**Fig. 5:**
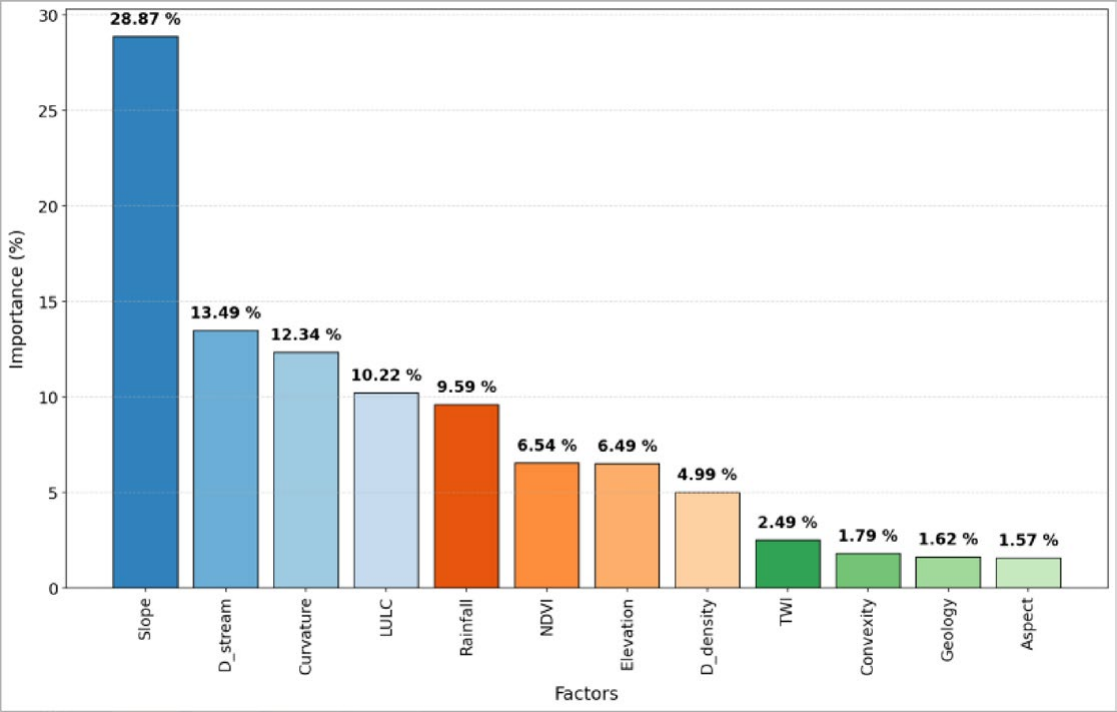
Average factor importance assessment using GWR method.

Results from GWR analysis (Fig. 5) showed that topographic and hydrological variables are the major controlling factors of flash flood occurrences. Slope, with 28.87%, was identified as the strongest predictor, followed by distance to streams and curvature, with 13.49% and 12.34%, respectively, featuring the importance of steepness of terrain, proximity to drainage, and local morphology for flood generation. Land use/land cover and rainfall contributed substantially with 10.22% and 9.59%, respectively, underlining their combined role due to surface conditions and climatic forcing. NDVI and elevation had a moderate influence, with 6.54% and 6.49%, respectively, whereas drainage density, TWI, convexity, geology, and aspect had minor influences, with 4.99%, 2.49%, 1.79%, 1.62%, and 1.57%, respectively. Overall, results confirm that flash flood susceptibility is principally determined by terrain configuration and hydroclimatic drivers, while vegetation, geology, and land cover play a secondary but moderating role.

**Fig. 6:**
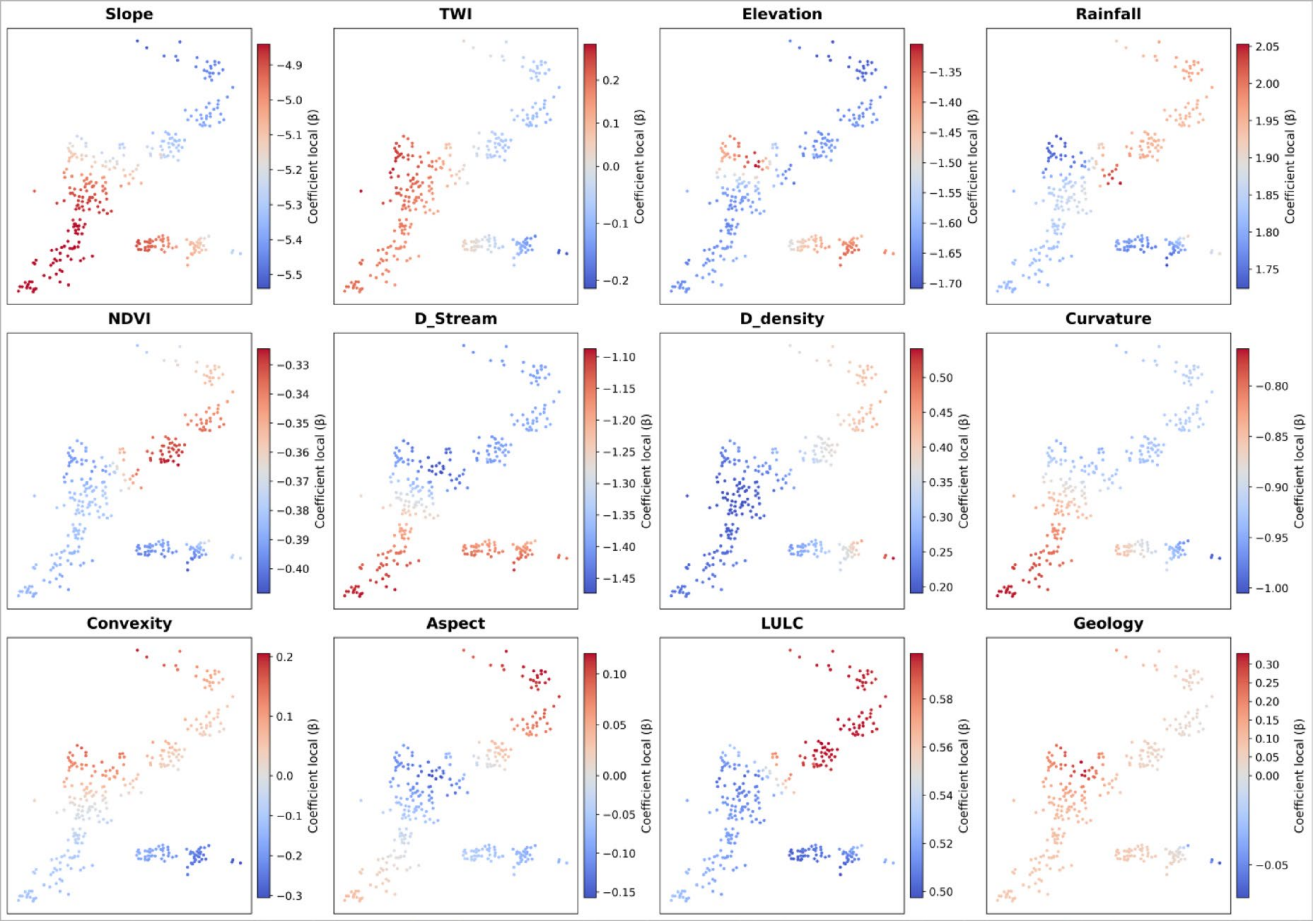
Spatial distribution of local coefficients (β) estimated by binomial GWR.

The local coefficient (β) maps derived from the binomial GWR presented in Fig. 6 show evident spatial non-stationarity in the role of flood-conditioning factors. Slope and distance to streams are the most relevant conditioning factors, since they have strong negative coefficients in all cases, thus increasing susceptibility in steep areas and near fluvial corridors. Rainfall and curvature also present high spatial variability, either as triggering or modulating factors, depending on local hydrological conditions. On the other hand, land use/land cover (LULC) and vegetation (NDVI) generally have a protective role in densely vegetated and agricultural areas. By contrast, drainage density, TWI, and convexity present more localized influences depending on fluvial concentration or dispersal processes, while elevation and aspect only impose residual and spatially differentiated effects. Geology is relevant only in some cases, depending on the permeability of the substrate. As a whole, these spatially very heterogeneous patterns confirm that the same variable might act as having opposite roles in different parts of the landscape, which again underlines the limits of global models and stresses the importance of geographically weighted methods to accurately characterise fine-scale flash flood-triggering processes .

### Variable importance analysis using random forest

**Fig. 7:**
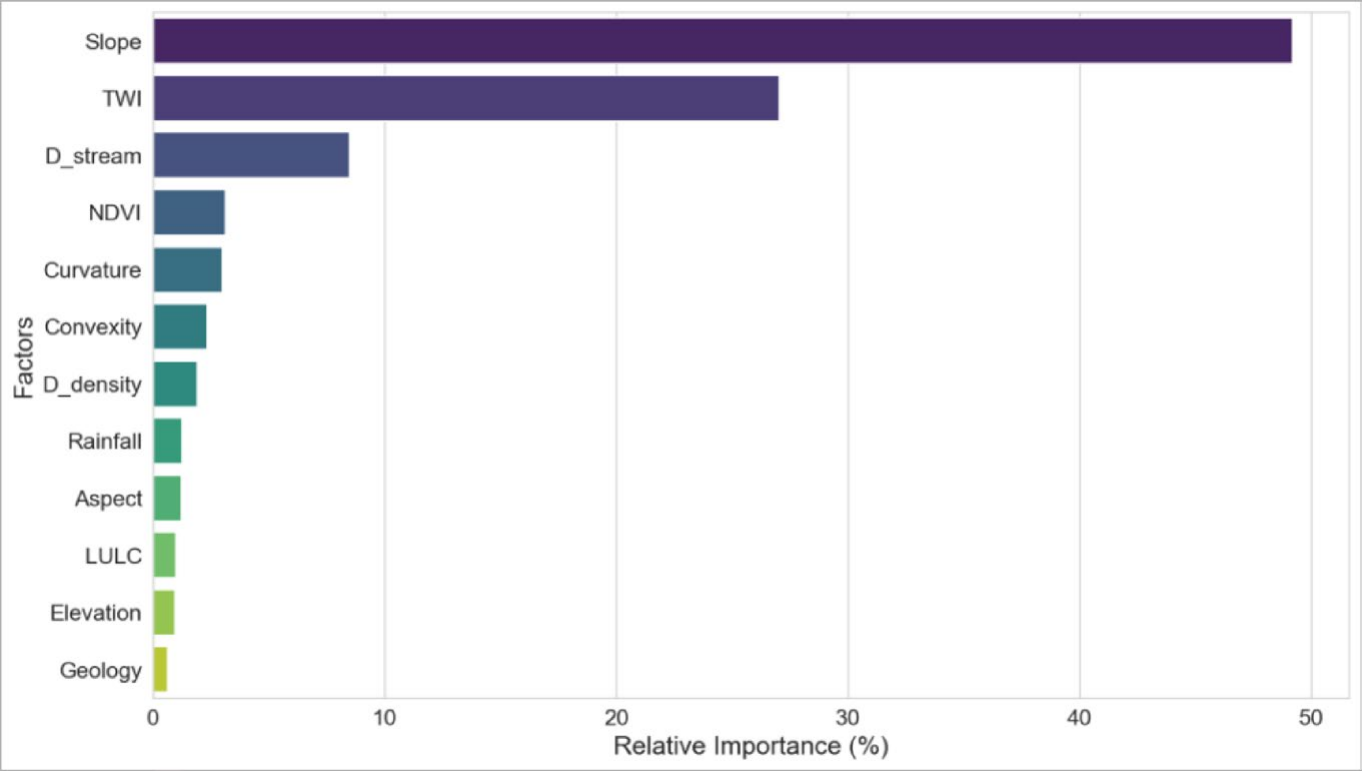
Average factor importance assessment using RF model.

The results of the variable importance analysis derived from the Random Forest (RF) model are presented in Fig. 7. The analysis identifies slope as the dominant controlling factor, accounting for 49.18% of the total importance, confirming its fundamental role in regulating surface runoff velocity and flow concentration. The Topographic Wetness Index (TWI) ranks second (27.03%), highlighting the influence of soil moisture accumulation and saturation processes in the generation of flash floods. Distance to the drainage network also shows a notable contribution (8.49%), indicating higher susceptibility in areas located close to streams.

Secondary contributions are associated with NDVI (3.12%), curvature (2.97%), convexity (2.32%), and drainage density (1.89%), suggesting that vegetation cover and terrain morphology exert a moderating effect on flash flood susceptibility. In contrast, rainfall (1.25%), land use/land cover (0.98%), elevation (0.94%), aspect (1.21%), and geology (0.62%) exhibit relatively low importance within the overall RF framework.

A comparison between the RF-based variable importance (Fig. 7) and the GWR-based assessment (Fig. 5) reveals a strong agreement in identifying the dominant controlling factors, particularly the predominance of topographic and hydrological variables. However, differences in the relative importance of certain factors, such as rainfall and land use, reflect the methodological distinctions between the two approaches. While GWR captures spatial non-stationarity and local variability (Fig. 6), the RF model provides a global, nonlinear assessment that emphasizes basin-scale structural controls.

### Convergence analysis and justification of optimization settings

**Fig. 8:**
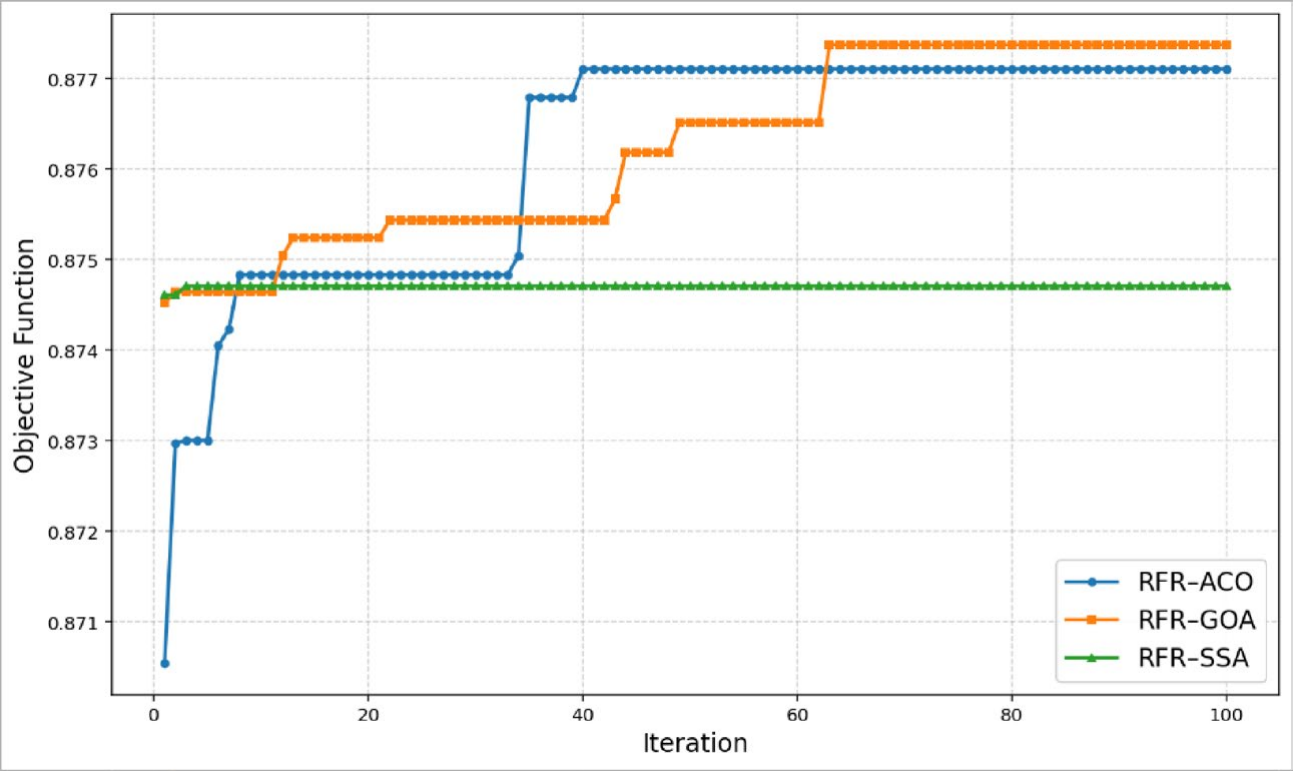
Convergence curves for the three metaheuristic optimizers.

Adequacy of the chosen optimization settings is shown by the convergence curves of the three metaheuristic optimizers presented in Fig. 8. The objective function used in the analysis corresponds to the mean coefficient of determination (R^2^) obtained through five-fold cross-validation, and the evaluations were performed using the testing dataset. Analysis of the obtained curves shows that all algorithms attain stable objective function values well before the maximum number of iterations. Specifically, the RFR-ACO model shows a gradual convergence with stabilization around 40 iterations, while the RFR-GOA model exhibits a longer exploration phase, converging at approximately 63 iterations. In contrast, the RFR-SSA model demonstrates rapid early convergence within the first few iterations, consistent with the behavior of exploitation-oriented swarm-based optimizers reported in recent metaheuristic reviews (e.g., comparisons of swarm intelligence algorithms and convergence behaviors)^140^.

Beyond these stabilization points, no significant improvement in the objective function is observed for any algorithm, indicating that 100 iterations are sufficient to achieve stable solutions without incurring unnecessary computational cost. Preliminary tests confirmed that increasing the iteration count beyond 100 did not yield measurable performance gains, in agreement with recent systematic analyses of metaheuristic optimization which highlight the importance of convergence assessment to avoid excessive iterations when the search space is relatively low-dimensional^141^.

Moreover, this optimization problem involves tuning a limited number of Random Forest hyperparameters, resulting in a comparatively low-dimensional search space where moderate population sizes have been shown to provide an effective balance between exploration and exploitation (as discussed in contemporary metaheuristic surveys)^142^. Accordingly, population sizes of 20 agents for ACO and 30 agents for SSA and GOA were adopted.

### Performance evaluation of established models

In the present investigation, the efficacy of the formulated Random Forest Regression (RFR) models, along with their metaheuristic-enhanced iterations, underwent thorough scrutiny via both numerical metrics and visual assessments. Optimization of the RFR framework was accomplished through the application of three distinct metaheuristic techniques: the Grasshopper Optimization Algorithm (GOA), Salp Swarm Algorithm (SSA), and Ant Colony Optimization (ACO), each calibrated with judiciously chosen control settings to promote swift convergence and dependable outcomes. As delineated in Table 4, a uniform iteration count of 100 was employed across all methods, whereas the agent population differed by algorithmic design: 30 grasshoppers in GOA, 30 salps in SSA, and 20 ants in ACO. Supplementary factors and constraints encompassing contraction coefficients for GOA and SSA, as well as pheromone and heuristic weighting elements for ACO were refined drawing upon established scholarly references and initial exploratory runs. Such configurations fostered an equitable interplay between exploratory and exploitative phases in the optimization sequence, thereby averting early stagnation and facilitating reliable refinement of the RFR’s hyperparameters.

**Table 4:** Metaheuristic algorithm parameters for model optimization.

Algorithms	Parameters
GOA	Number of iterations = 100
	Number of grasshoppers = 30
	Maximum contraction coefficient (c_max_) = 1.0
	Minimum contraction coefficient (c_min_) = 0.00001
	Lowerbounds = [10, 0.1, 5]
	Upperbounds = [100, 1.0, 30]
SSA	Number of iterations = 100
	nNumber of salps = 30
	Maximum contraction coefficient (c_max_) = 2
	Minimum contraction coefficient (_Cmin_) = 0
	Lowerbounds = [10, 0.1, 5]
	Upperbounds = [100, 1.0, 30]
ACO	Number of iterations = 100
	Number of ants = 20
	Evaporation rate = 0.1
	Influence of pheromone (α) = 1
	Influence of heuristic (β) = 2
	Lowerbounds = [10, 0.1, 5]
	Upperbounds = [100, 1.0, 30]

The optimization process centered on three pivotal hyperparameters of the Random Forest model: the number of trees (n_estimators), the fraction of features considered (max_features), and the maximum tree depth (max_depth), all of which profoundly influence the model’s architectural intricacy and its ability to generalize effectively. As outlined in Table 5, the refined configurations reveal that the metaheuristic algorithms arrived at varied yet mutually supportive optima. The GOA-optimized variant utilized 98 trees, a feature selection ratio of 0.68, and a depth of 13.6, whereas the ACO-optimized model incorporated 93 trees, a max_features setting of 0.69, and a depth of 14.4. In contrast, the SSA-optimized approach opted for a more profound structure with a depth of 16, complemented by a reduced number of trees (78.5) and an elevated feature proportion (0.99). Such findings imply that GOA and ACO tended toward concise and resilient frameworks, while SSA prioritized an expansive examination of feature interrelations, thereby potentially elucidating more intricate dependencies within the hydrological variables.

**Table 5:** Optimized hyperparameters of the RFR model.

Model	Hyperparameter	GOA	ACO	SSA
RFR	*n_estimators*	98.36	93.32	78.51
	*max_features*	0.68	0.69	0.99
	*max_depth*	13.63	14.38	16.23

The efficacy of the baseline and enhanced Random Forest Regressor (RFR) models was appraised via conventional evaluative measures, such as the coefficient of determination (R^2^), mean squared error (MSE), mean absolute error (MAE), and root mean squared error (RMSE), as detailed in Table 6. On the validation dataset, the unoptimized RFR model registered an R^2^ of 0.8445 and an RMSE of 0.1491, signifying adequate but limited predictive prowess alongside evident overfitting, as evidenced by its elevated training R^2^ of 0.9592. Post-optimization, each metaheuristic-augmented variant displayed marked advancements in performance and enhanced capacity for generalization. Specifically, the RFR-GOA attained an R^2^ of 0.9767, MSE of 0.0009, MAE of 0.0215, and RMSE of 0.0292, illustrating a commendable equilibrium between bias and variance. The RFR-SSA yielded analogous outcomes (R^2^ = 0.9548, MSE = 0.0017, MAE = 0.0310, RMSE = 0.0416), affirming reliable convergence and steadfast forecasting capabilities. Outperforming the others, the RFR-ACO achieved the pinnacle of results with an R^2^ of 0.9781, MSE of 0.0008, MAE of 0.0215, and RMSE of 0.0290, denoting exceptional predictive fidelity and an ideal harmony between training and validation efficacy. Collectively, these enhancements substantiate the prowess of metaheuristic techniques in precisely calibrating the Random Forest’s hyperparameters, thereby alleviating overfitting concerns.

**Table 6:** Performance results of the developed models.

Models	Testing				Training			
	R^2^	MSE	MAE	RMSE	R^2^	MSE	MAE	RMSE
RFR	0.8445	0.0222	0.1093	0.1491	0.9592	0.0077	0.0696	0.0876
RFR-GAO	0.9767	0.0009	0.0215	0.0292	0.9838	0.0006	0.0190	0.0254
RFR-SSA	0.9548	0.0017	0.0310	0.0416	0.9639	0.0014	0.0292	0.0379
RFR-ACO	0.9781	0.0008	0.0215	0.0290	0.9842	0.0006	0.0188	0.0251

**Fig. 9:**
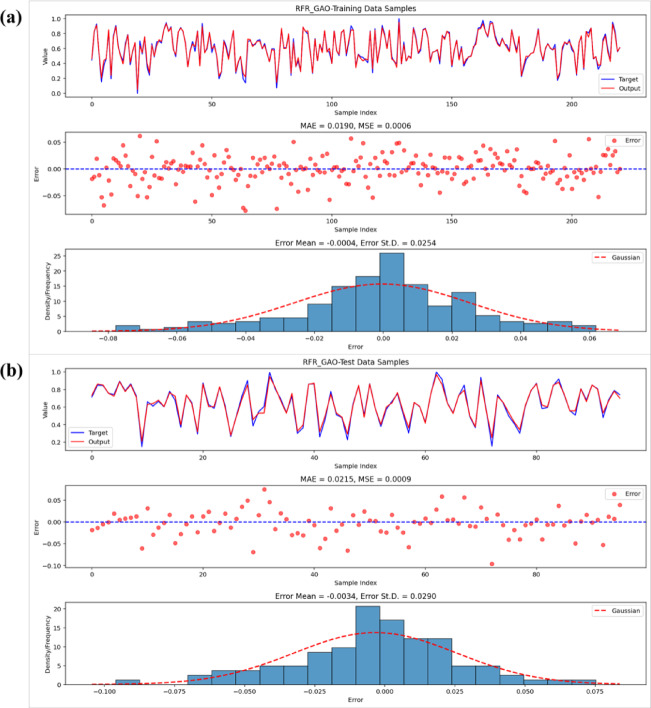
Performance of the RFR-GOA model: observed vs. predicted values, errors, and residuals for (**a**) training and (**b**) test data.

**Fig. 10:**
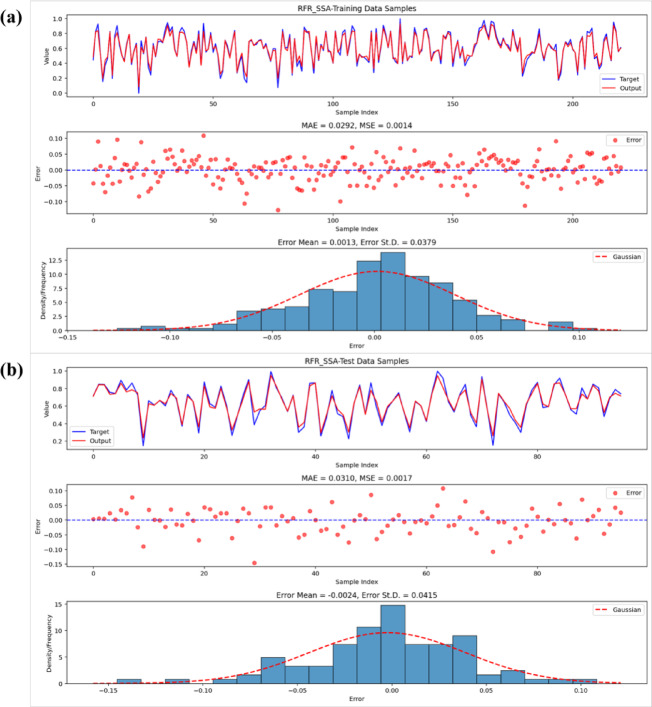
Performance of the RFR-SSA model: observed vs. predicted values, errors, and residuals for (**a**) training and (**b**) test data.

**Fig. 11:**
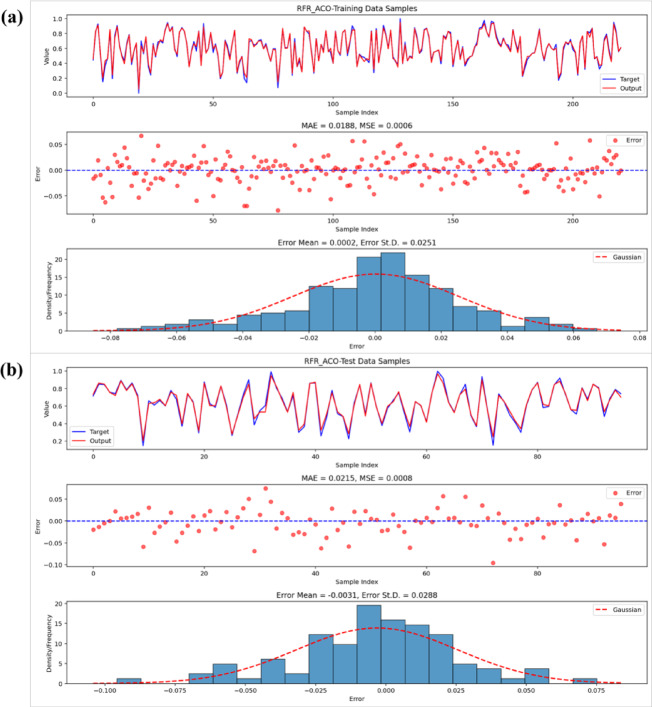
Performance of the RFR-ACO model: observed vs. predicted values, errors, and residuals for (**a**) training and (**b**) test data.

Visual inspections detailed in Figs. 9, 10 and 11 bolster the quantitative results through an exploration of residual patterns, the congruence between predicted and observed values, and the spread of errors. In the case of the RFR-GOA model, residuals displayed a distribution approximating Gaussian form, clustered tightly around zero with minimal bias and limited variability, which points to stochastic variations rather than patterned inconsistencies. The strong alignment of observed and forecasted outcomes across training and validation stages further attests to the model’s computational robustness and capacity for effective extrapolation. In a parallel vein, the RFR-SSA model’s residuals showed symmetric dispersion coupled with a modest standard deviation (ranging from 0.038 to 0.042), underscoring the SSA method’s aptitude for discerning complex, nonlinear hydrological interactions while upholding steady predictive dependability. The RFR-ACO residuals, by comparison, were yet more concentrated and precisely aligned, manifesting an almost ideal Gaussian profile with trifling average errors (on the order of −0.003 in the testing set), thereby establishing ACO as the optimizer that yielded the most dependable and variance-homogeneous setup among those assessed. Taken together, these observations affirm that the metaheuristic strategies adeptly generated impartial and finely tuned RFR models, devoid of entrenched biases or exaggerated variance.

**Fig. 12:**
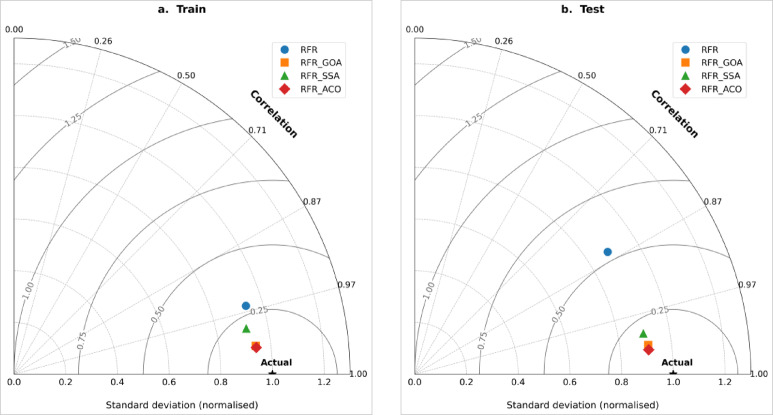
Taylor diagram results for the four models: (**a**) training and (**b**) testing datasets.

The Taylor diagrams (Fig. 12) offer a concise visualization of the models’ relative performance, concurrently illustrating the correlation coefficient and normalized standard deviation in relation to empirical observations. The trio of metaheuristic-optimized models congregate in close proximity to the “Actual” benchmark, signifying strong correlations and minimal variability in errors, in stark contrast to the baseline RFR, which is positioned more distantly from this reference, thereby exposing reduced reliability and heightened variability. Within the optimized group, the RFR-ACO model resides nearest to the benchmark, with RFR-SSA and RFR-GOA trailing in sequence, which affirms that ant colony optimization yielded the optimal equilibrium of accuracy and robustness. Furthermore, the akin placements of RFR-GOA and RFR-SSA imply that these algorithms attained configurations approaching optimality, with their distinctions largely attributable to variations in the balance of depth features within the ensuing Random Forest Regressor architectures.

Synthesizing the insights derived from quantitative metrics and visual evaluations establishes a clear ranking of model efficacy: RFR-ACO surpassing RFR-GOA, which is roughly equivalent to RFR-SSA, with all three markedly outperforming the baseline RFR. The incorporation of metaheuristic algorithms significantly augmented the Random Forest Regressor’s proficiency in discerning both linear and nonlinear associations across environmental and hydrological variables, thereby yielding predictions of flood susceptibility that are both more precise and broadly applicable. Notably, the ACO method delivered the most resilient optimization, achieving superior accuracy alongside minimized error rates, whereas GOA and SSA contributed equivalent yet synergistic advantages in terms of convergence speed and adaptive model architecture.

### Flood susceptibility mapping using developed models

**Fig. 13:**
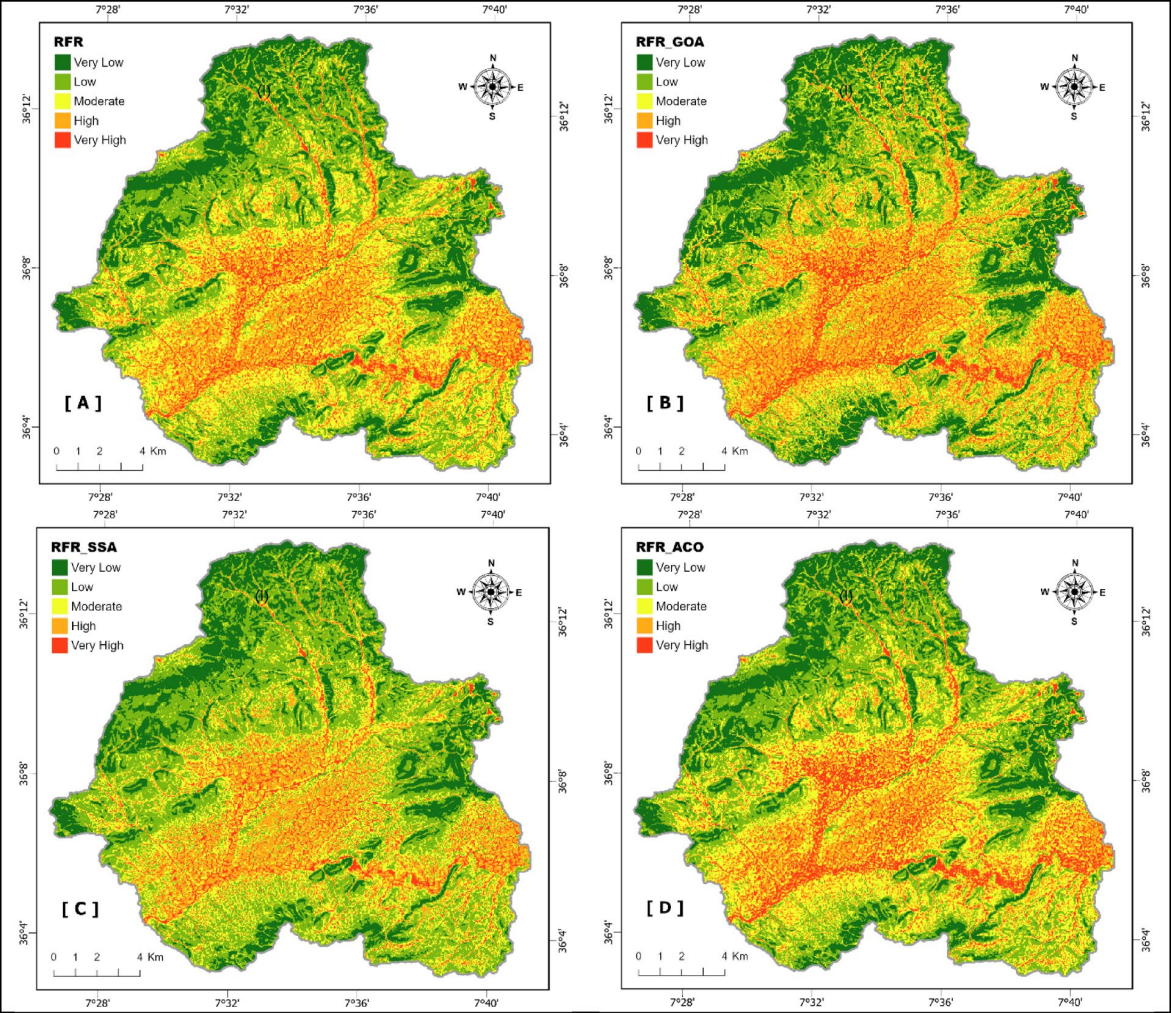
Flood susceptibility maps of the SedrataWatershed generated using four machine learning models: (**A**) Random Forest Regressor (RFR), (**B**) RFR optimized by the Grasshopper Optimization Algorithm (RFR-GOA), (**C**) RFR optimized by the Salp Swarm Algorithm (RFR-SSA), and (**D**) RFR optimized by the Ant Colony Optimization Algorithm (RFR-ACO).

Flood susceptibility assessment for the Sedrata Basin (Fig. 13) was conducted employing the Random Forest Regressor (RFR) model alongside three metaheuristic-enhanced versions: RFR-GOA, RFR-SSA, and RFR-ACO. The pixel-based continuous susceptibility scores were categorized into five levels, very low, low, moderate, high, and very high, utilizing the natural breaks (Jenks) classification approach. The produced maps demonstrate substantial spatial agreement among the models, underscoring the inherent reliability of the RFR algorithm in forecasting flood vulnerability, irrespective of the applied optimizations. Regions classified as high or very high susceptibility are predominantly situated in the basin’s central, southern, and eastern regions, aligning with areas featuring low elevations, mild gradients, extensive drainage networks, and hydrologic attributes conducive to surface water buildup. Conversely, the northern and northwestern areas, marked by sharper inclines and enhanced soil permeability, generally display susceptibility ratings ranging from low to very low.

**Fig. 14:**
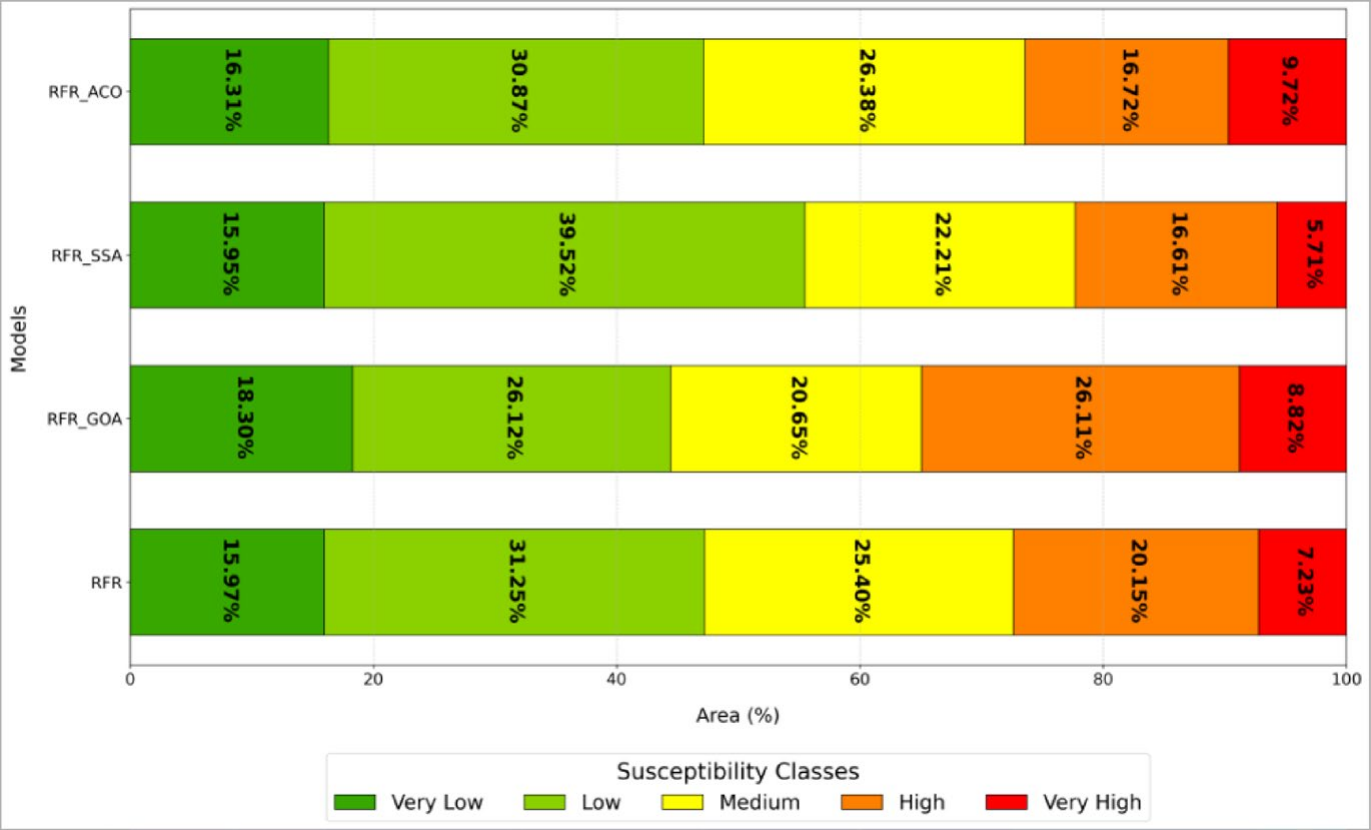
Land area distribution by five flood vulnerability classes for the four models.

While the models exhibit broadly comparable spatial distributions, notable variations emerge upon closer examination. The RFR-ACO model delineates more expansive zones of high and very high flood susceptibility, especially within the central region of the basin, in contrast to the more restrained classifications offered by the RFR-SSA and RFR-GOA models. These variations stem from the unique proficiencies of each metaheuristic algorithm in navigating and capitalizing on the parameter space to refine predictive outcomes. Broadly speaking, regions of low and moderate susceptibility predominate, covering 55–60% of the basin’s area, whereas high and very high susceptibility zones constitute 25–35%. In particular, the RFR-ACO model assigns the greatest share to very high susceptibility areas, at 9.72%. (Fig. 14).

### Validation of flood susceptibility maps

**Fig. 15:**
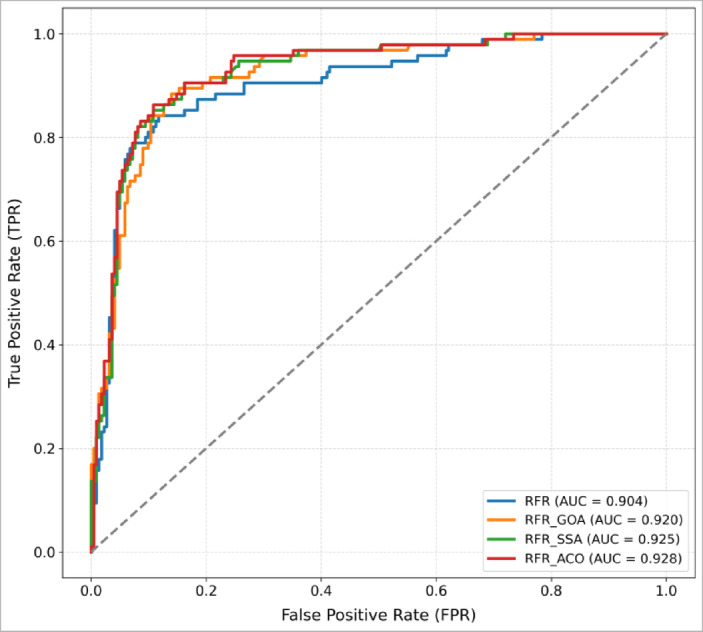
ROC curve evaluation of the four established models.

In this study, the performance of the developed models was assessed through a range of error metrics, including mean squared error (MSE), mean absolute error (MAE), root mean squared error (RMSE), and the coefficient of determination (R^2^), alongside an analysis of the receiver operating characteristic (ROC) curve (Fig. 15). The model exhibiting the greatest area under the curve (AUC) is regarded as having superior predictive efficacy. Based on the AUC outcomes, the RFR model optimized with ant colony optimization (RFR_ACO) attained the highest performance(0.928), with RFR_SSA (0.925) and RFR_GOA (0.920) following closely behind. In contrast, the unoptimized baseline RFR model yielded the lowest AUC of 0.904. Notably, all models surpassed an AUC threshold of 0.9, reflecting outstanding capabilities in mapping flood susceptibility. These ROC comparisons underscore how incorporating metaheuristic optimization techniques such as ACO, SSA, and GOA bolstered the Random Forest Regressor’s predictive power. Of these approaches, ACO emerged as the most effective, delivering the greatest enhancement in overall model accuracy. Consequently, the models can be ranked in descending order of performance as follows: RFR-ACO > RFR-SSA > RFR-GOA > RFR.

## Discussion

### Analysis and interpretation of spatial data

Integrating GWR and WoE approaches provided deep insights into the spatial controls on flood susceptibility. According to the GWR model outputs, slope, drainage density, and curvature are the most dominant factors, collectively accounting for the lion’s share of the spatial variability within flood events. These factors reflect the geomorphological controls over runoff concentration in semi-arid basins^31^. Complementing these findings, the WoE outputs identified lower slopes (0–2%), streams proximity (< 200 m), and a low vegetation index (NDVI < 0.2) as critical thresholds that exhibit maximum flood occurrence, confirming results obtained in Mediterranean and North African basins by Loukili et al^38^. and Wahba et al^32,33^., respectively. Furthermore, the non-stationarity coefficients observed from GWR confirm that susceptibility modeling cannot be done without considering spatial heterogeneity, as stated by Fotheringham et al^111^. Therefore, the coupling of spatially explicit models and interpretable machine learning methods strengthens physical credibility with policy relevance in generating flood risk maps.

### Performance and evaluation of the developed models

The metaheuristic-optimized Random Forest models significantly outperformed the baseline model, with RFR-ACO producing the best performance among them (R^2^ = 0.978; RMSE = 0.029). This agrees with recent research indicating that bioinspired optimization techniques, such as ACO, GOA, and SSA, enhance the generalization capacity of machine learning models in flood predictions^46,47,49,50^. The gains are realized in the balance between exploration and exploitation in parameter searching for each of the optimizers^118,126^. Also, the ACO-based model performed the best due to its capability for efficient exploration in the high-dimensional parameter space through pheromone feedback, thus confirming similar trends found by Taranto et al^129^.. Moreover, the incorporation of Sentinel-1 imagery increased the spatial coherence, in agreement with the work done by Breznik et al^34^., where SAR-based flood detection was able to provide reliability comparable to hydraulic models. Overall, our hybrid framework supports recent research advances in multisourced data-driven flood modeling^51^ and strengthens the potential of optimized ensemble schemes for environmental prediction.

Several hybrid approaches combining machine-learning models with metaheuristic algorithms have been proposed for flood susceptibility analysis. For example, Hoang and Liou^60^ optimized SVM, RF and XGBoost using Particle Swarm Optimization and Genetic Algorithans for flash-flood susceptibility mapping, and showed that swarm-based and evolutionary optimizers significantly improve the predictive skill of these models compared to their non-optimized counterparts. Another recent study incorporated a Grey Wolf Optimizer (GWO) into various machine learning models (including XGBoost and SVR), showing that GWO-enhanced models achieved higher predictive accuracy compared to the unoptimized versions, and highlighting the added value of metaheuristic optimization in flood susceptibility modelling^49^. More recently, Razavi-Termeh et al^46,47^. developed a synergistic framework in which the CatBoost boosting algorithm is optimized by two swarm-based metaheuristics (Whale Optimization Algorithm and Zebra Optimization Algorithm) for flood-prone area mapping, achieving notable gains in accuracy relative to the non-optimized CatBoost model.

Compared to these contemporary hybrid metaheuristic-based studies, the present research advances the state of the art by systematically integrating a Random Forest Regressor with three distinct metaheuristic algorithms (GOA, SSA and ACO) within a unified framework, enabling a direct comparison of their relative benefits, and evaluating not only predictive performance but also the convergence behavior and optimization stability of each optimizer. Moreover, this hybrid framework was applied to a semi-arid North African watershed using a Sentinel-1-derived flood inventory, a regional and data context that is still rarely considered in hybrid ML-metaheuristic flood susceptibility research. In addition, by combining global modeling using RF with local spatial analysis via Geographically Weighted Regression (GWR), our approach delivers both enhanced predictive accuracy and improved interpretability of conditioning factors, addressing limitations observed in other hybrid studies.

### Study limitations and research perspectives

Despite the encouraging results obtained, two primary limitations remain. First, the currently adopted models use static conditioning factors, while flood dynamics change over time, considering rainfall intensity, antecedent soil moisture, and land use changes. Incorporating time-series data and dynamic hydrological indices can enhance model temporal adaptability. Moreover, the static approach followed in this research may be updated by adopting conditional random fields, as pointed out by Sheikh and Coulibaly^143,144^and Breznik et al^34^.. On the other hand, Sentinel-1 backscatter data might bring ambiguities due to the surface roughness effects in urban or vegetated areas. Future research should combine multi-temporal SAR data, such as Sentinel-1, RADARSAT, TerraSARX data, together with real-time hydrometeorological information for enhancing the accuracy.

Although the set of twelve conditioning factors captures the main topographic, hydrometeorological and land‑use controls on flood processes in the Sedrata Watershed, some important terrain‑related variables such as soil type, soil texture and permeability could not be incorporated due to the lack of consistent, high‑resolution datasets for the study area. This omission is a clear limitation of the present work, as soil properties strongly influence infiltration capacity, runoff generation and, consequently, the spatial pattern of flood susceptibility. The resulting maps should therefore be interpreted as conditional on the available geospatial information, and future research should aim to integrate harmonized soil and permeability data from regional surveys or global databases in order to further improve the physical realism and local‑scale accuracy of the susceptibility assessment.

To enhance the model interpretability, integrating explainable Artificial Intelligence (AI) such as SHapley Additive exPlanations (SHAP) or Local Interpretable Model-agnostic Explanations (LIME) in the modeling framework can improve the interpretability of the models in the identification of variable importance^48^. The performance of hybrid deep learning and optimization methods may yield better spatial generalization^46,47^. The analysis will ultimately rely on balanced performance metrics, particularly the Matthews Correlation Coefficient (MCC), which provides an unbiased evaluation under class imbalance conditions^139^. From a scientific and policy perspective, the optimized RFR-ACO model achieved AUC values exceeding 0.928, indicating high predictive accuracy. The resulting flood susceptibility maps provide reliable and actionable insights to support local-scale flood risk reduction planning and decision-making.

High-risk zones, characterized by low elevations and high drainage densities, should be prioritized for infrastructure strengthening, floodplain rehabilitation measures, and the deployment of nature-based solutions. This is particularly significant for urbanized expansions along flood-prone corridors in Mediterranean and semi-arid basins^31,51^. The Sentinel-1-driven methodological framework integrating spatially explicit modeling and optimized machine learning forms a transferable and cost-effective instrument for risk management over data-sparse regions. On scientific grounds, this study demonstrates the pivotal role played by the combination of physically interpretable predictors and data-driven optimization methods to bridge the gap between predictive accuracy and hydrological realism. These findings fit within ongoing state-of-the-art priorities concerned with sustainable flood management and spatial decision-support systems^38,111^.

## Conclusions

Hybrid models proposed in this study, considered as a decision-aid tool, yielded reasonably good results with Area Under Curve (AUC) values varying between 0.92 and 0.93 and correlation coeffficients exceeding 0.95. Compared to the baseline model, the integration of metaheuristic algorithms in the Random Forest model significantly increased its predictive capacity and model stability. The spatial patterns identified by the models show that those with gentle slopes, low elevation, low NDVI, a high topographic wetness index, and with dense drainage networks fall within a zone of high flood susceptibility. These zones are distributed throughout most of the central and downstream parts of the watershed and correspond to areas of intensive agricultural and urban activities.

The results obtained are quite interesting and useful for sustainable land and water management in flood-prone areas. Nevertheless, the absence of detailed soil and permeability information represents a limitation of the current framework, and integrating such datasets in future work is expected to further refine the flood susceptibility patterns, especially at local scales. Moreover, the proposed models use static conditioning factors, while flood dynamics, affected by rainfall intensity, antecedent soil moisture, and land use, change over time. Finally, it is important to note that future research may be directed towards exploring more metaheuristic algorithms, deep learning models, and explainable AI techniques to enhance model interpretability and real-time applicability. Besides, integration with web-based, mobile, and IoT platforms will make this framework functional and effective to serve the needs of decision-makers and urban planners interested in flood risk reduction and climate adaptation.

## Data Availability

The data that support the findings of this study are not publicly available due to institutional restrictions but are available from the corresponding author upon reasonable request.
